# A Novel Motif in the 3′-UTR of PRRSV-2 Is Critical for Viral Multiplication and Contributes to Enhanced Replication Ability of Highly Pathogenic or L1 PRRSV

**DOI:** 10.3390/v14020166

**Published:** 2022-01-18

**Authors:** Junyao Xiong, Xingyang Cui, Kuan Zhao, Qian Wang, Xinyi Huang, Dongyan Li, Fang Yu, Yongbo Yang, Di Liu, Zhijun Tian, Xuehui Cai, Tongqing An

**Affiliations:** 1 State Key Laboratory of Veterinary Biotechnology, Harbin Veterinary Research Institute, Chinese Academy of Agricultural Sciences, Harbin 150069, China; xiongjunyao@foxmail.com (J.X.); cxy_2010@126.com (X.C.); zhaokuan519@126.com (K.Z.); qianwang919@163.com (Q.W.); huangxinyi683@163.com (X.H.); ldy1765696750@163.com (D.L.); 15733239763@163.com (F.Y.); yangyongbo@caas.cn (Y.Y.); tianzhijun@caas.cn (Z.T.); caixuehui@caas.cn (X.C.); 2 Institute of Animal Husbandry, Heilongjiang Academy of Agricultural Sciences, Harbin 150086, China; liudi1963@163.com

**Keywords:** HP-PRRSV, 3′-UTR, secondary structure, motif, replication, epidemic

## Abstract

Highly pathogenic porcine reproductive and respiratory syndrome virus (HP-PRRSV) with enhanced replication capability emerged in China and has become dominant epidemic strain since 2006. Up to now, the replication-regulated genes of PRRSV have not been fully clarified. Here, by swapping the genes or elements between HP-PRRSV and classical PRRSV based on infectious clones, NSP1, NSP2, NSP7, NSP9 and 3′-UTR are found to contribute to the high replication efficiency of HP-PRRSV. Further study revealed that mutations at positions 117th or 119th in the 3′-UTR are significantly related to replication efficiency, and the nucleotide at position 120th is critical for viral rescue. The motif composed by 117–120th nucleotides was quite conservative within each lineage of PRRSV; mutations in the motif of HP-PRRSV and currently epidemic lineage 1 (L1) PRRSV showed higher synthesis ability of viral negative genomic RNA, suggesting that those mutations were beneficial for viral replication. RNA structure analysis revealed that this motif maybe involved into a pseudoknot in the 3′-UTR. The results discovered a novel motif, 117–120th nucleotide in the 3′-UTR, that is critical for replication of PRRSV-2, and mutations in the motif contribute to the enhanced replicative ability of HP-PRRSV or L1 PRRSV. Our findings will help to understand the molecular basis of PRRSV replication and find the potential factors resulting in an epidemic strain of PRRSV.

## 1. Introduction

Porcine reproductive and respiratory syndrome (PRRS) is one of the most important infectious diseases in the pig industry, characterized by severe reproductive failure in sows and respiratory diseases in all-age pigs [1]. It was first reported in the United States in 1987 [2], and it is currently found in all major pig-raising countries [3,4,5,6]. The etiological agent, PRRS virus (PRRSV), is an enveloped, single-stranded and positive-sense RNA virus. The PRRSV genome encodes at least 11 open reading frames (ORFs), flanked by 5′-UTR (untranslated region, UTR) and 3′-UTR [7,8,9]. ORF1a and ORF1b encode polyproteins of PP1a and PP1ab, in which at least 16 nonstructural proteins (NSPs) are produced after hydrolysis and cleavage [10,11,12,13,14,15,16,17]. Meanwhile, ORF2a, ORF2b, ORF3, ORF4, ORF5, ORF5a, ORF6 and ORF7 encode the structural proteins of GP2a, E, GP3, GP4, GP5, ORF5a, M and N, respectively [9,18].

PRRSV is a member of the *Arteriviridae* family in the *Nidovirales* order (including *Coronaviridae* family, *Arteriviridae* family and *Roniviridae* family). Viruses in these families have very different virion morphology, but the grouping reflects their common and distinctive replication strategy that utilizes a nested set of 3′ co-terminal subgenomic messenger RNA (sgmRNA). During the period of PRRSV replication, RNA-dependent RNA polymerase (RdRp) and RNA helicase, encoded by NSP9 and NSP10, respectively, together with other NSPs (including NSP2, NSP3 and NSP5) assemble into a replication and transcription complex (RTC) [19,20]. Eventually, RTC binds to the 3′-UTR and initiates viral negative-strand RNA synthesis, which further regulates the sgmRNA transcription. The synthesized interfering RNA targeted to the PRRSV 3′-UTR could significantly decrease the transcription of sgmRNA and viral genome replication [21]. The importance of 3′-UTR is also confirmed in other Arterivirus or Coronaviruses, such as mouse hepatitis virus (MHV), equine arteritis virus (EAV), porcine epidemic diarrhea virus (PEDV) and Chikungunya virus [22,23,24,25]. However, the key sites or motifs of PRRSV 3′-UTR regulating viral replication have not been clarified.

PRRSV is classified into two genotypes, European-type PRRSV (PRRSV-1) and North American type (PRRSV-2) PRRSV, represented by LV strain and VR-2332 strain, which share approximately 60% nucleotide similarity [26]. The first Chinese PRRSV, CH-1a strain, was reported in 1996 [27]. Through the gradual evolution of CH-1a-like PRRSV [28], a high pathogenic PRRSV variant (HP-PRRSV) appeared in 2006 in China, resulting in more than 200 million deaths in pigs. Soon afterward, HP-PRRSV was reported in about ten Asian countries [29] and also became an absolutely dominant epidemic strain in China [30]. In 2013, NADC30-like PRRSV strain was imported from North America to China [31], which spread quickly and is a currently dominant strain in China [5].

Compared to classical PRRSV, HP-PRRSV exhibits higher replication efficiency in vivo and in vitro [32,33], but the viral genes or elements that affect the level of viral replication are unknown. In this study, based on the infectious clones of classical PRRSV CH-1a strain and HP-PRRSV HuN4 strain, a series of chimeric viruses was constructed by swapping the corresponding genes or elements, and the results showed that 3′-UTR and several NSPs are closely related to the replication level of HP-PRRSV. Moreover, a motif in the 3′-UTR is found to be critical for the replication of HP-PRRSV, and mutations in the motif enhance the level of viral negative-strand RNA synthesis of NADC30-like PRRSV. Our findings may help to understand the epidemic of HP-PRRSV or NADC30-like PRRSV.

## 2. Materials and Methods

### 2.1. Cells and Viruses

Marc-145 cell was cultured in Dulbecco’s modified Eagle’s medium (DMEM, Sigma-Aldrich, St. Louis, MO, USA), supplemented with 10% fetal bovine serum (FBS, Excell), 100 IU/mL penicillin and 100 µg/mL streptomycin at 37 °C with 5% CO_2_. Porcine alveolar macrophages (PAMs), the primary target cell for PRRSV, was prepared from lung lavage of 3-week-old to 4-week-old specific pathogen-free piglets. PAMs were cultured in RPMI-1640 medium (Gibco, Thermo Fisher Scientific, Waltham, MA, USA). The infectious clones, rescued viruses of HP-PRRSV HuN4 strain (GenBank no. EF635006) and classical PRRSV CH-1a strain (GenBank no. AY032626) were preserved in our laboratory.

### 2.2. Genetic Analysis of 3′-UTR Sequences of PRRSV-2

All available PRRSV-2 3′-UTR sequences (*n* = 765) from 1992 to 2019 were retrieved from GenBank (Table S1). Its isolation places included ten countries: China, USA, Canada, Denmark, Hungary, Japan, Laos, South Korea, Thailand and Vietnam. Multiple sequence alignment was carried out using Lasergene software^®^ (DNASTAR, Madison, WI, USA). The phylogenetic tree was generated by ML method using MEGA 7 [34], and bootstrap values were calculated on 1000 replicates. The classification of lineages was performed according to previous descriptions [35,36].

### 2.3. Prediction of RNA Secondary Structure

The RNA secondary structure was predicted by the mFold website server for nucleic acid folding and hybridization prediction (http://www.unafold.org/, accessed on 11 November 2020) under default folding parameters (37 °C, 1 M NaCl, no divalent ions and no limit on the distance between paired bases) [37]. The predicted secondary structure was modified by the RNAviz 2.0 software (http://rnaviz.sourceforge.net/, accessed on 11 November 2020) [38]. According to previous studies, the predicted pseudoknot in the 3′-UTR was depicted. [22,23,39,40,41,42,43].

### 2.4. Construction and Rescue of Chimeric or Mutant Viruses

Based on the infectious clones of HuN4 or CH-1a, a series of chimeric plasmids of pHC_1_, pCH_1_, pHC_2_, pCH_2_, pHC_3_, pCH_3_, pHC_4_, pCH_4_, pHC_5_, pCH_5_, pHC_7_, pCH_7_, pHC_9_, pCH_9_, pHC_10_, pCH_10_, pHC_11_, pCH_11_, pHC_12_, pCH_12_, pHC_GP2_, pCH_GP2_, pHC_GP3_, pCH_GP3_, pHC_GP4_, pCH_GP4_, pHC_GP5_, pCH_GP5_, pHC_M_, pCH_M_, pHC_3′-UTR_ and pCH_3′-UTR_ were constructed, which swapped the corresponding regions of NSP1, NSP2, NSP3, NSP4, NSP5, NSP7, NSP9, NSP10, NSP11, NSP12, GP2, GP3, GP4, GP5, M and 3′-UTR, respectively. Moreover, in order to find the key nucleotides of 3′-UTR, a series of mutant plasmids of pHuN4-3′-UTR-C9T, pHuN4-3′-UTR-17+G, pHuN4-3′-UTR-A117G, pHuN4-3′-UTR-A119G, pHuN4-3′-UTR-G120A, pHuN4-3′-UTR-117+120, pHuN4-3′-UTR-119+120 and pHuN4-3′-UTR-117+119+120 was constructed by site-directed mutagenesis. In brief, all mutant plasmids were introduced into HuN4 infectious clone by site-directed PCR-based mutagenesis. Firstly, PCR products were amplified by using different mutant primers with the template of HuN4. Secondly, the varied PCR products of the same mutation were fused by fusion PCR. Then, HuN4 plasmid and mutant DNA fragments were digested with the same restriction enzyme of *Mlu* I and *Pac* I. Finally, the HuN4 vector and the mutant DNA fragments were ligated by DNA ligase, and the desired mutant plasmids were constructed successfully. All plasmids were subject to nucleotide sequencing. All primers were listed in Table 1.

For viral rescue, Marc-145 cells with 80% confluence seeded in 6-well plates were transfected with the chimeric, mutant or backbone plasmids using X-tremeGENE HP DNA transfection reagent (Roche, Switzerland) according to the manufacturer’s instruction. The rescued chimeric viruses were individually designated HC_1_, CH_1_, HC_2_, CH_2_, HC_3_, CH_3_, HC_4_, CH_4_, HC_5_, CH_5_, HC_7_, CH_7_, HC_9_, CH_9_, HC_10_, CH_10_, HC_11_, CH_11_, HC_12_, CH_12_, HC_GP2_, CH_GP2_, HC_GP3_, CH_GP3_, HC_GP4_, CH_GP4_, HC_GP5_, CH_GP5_, HC_M_, CH_M_, HC_3′-UTR_ and CH_3′-UTR_, respectively. The rescued 3′-UTR mutant viruses were individually designated HuN4-3′-UTR-C9T, HuN4-3′-UTR-17+G, HuN4-3′-UTR-A117G and HuN4-3′-UTR-A119G, respectively. The supernatants of the transfected cells were harvested at 96 h post-transfection, designated as passage 1 (P1) of the rescued viruses. P1 was continuously passaged to the third generation (P3), followed by viral titration on Marc-145 cells, and the viruses were stored at −80 °C for later use. All viruses (P3) were subject to nucleotide sequencing.

### 2.5. Viral Titration and Growth Kinetics

For viral titration, confluent Marc-145 cells seeded in 96-well plates were incubated with 10-fold serially diluted viral suspensions, and the viral titers were measured according to the method of Reed and Muench [44]. Briefly, the confluent Marc-145 cells seeded in 96-well plates were incubated with 10-fold serially diluted viral suspensions. After absorption for 1 h at 37 °C, the supernatants were removed, and the fresh DMEM with 2% FBS was added. Following this, the plates were incubated for an additional six days, and viral titers were determined by the presence of visible cytopathic effect (CPE). Finally, the viral titers were calculated according to the method of Reed and Muench.

In order to investigate the replication difference of rescued mutant viruses and their parental viruses (rescued HuN4 or CH-1a), confluent Marc-145 cells or PAMs were incubated with the mutant and parental viruses at a multiplicity of infection (MOI) of 0.01. After inoculation for 1 h at 37 °C, the supernatants were discarded, and cells were washed with PBS three times. Following this, fresh DMEM with 2% FBS was added. At last, cell supernatants were harvested at 0, 24, 48, 72 and 96 h post-infection (hpi). The viral titers of different time points were measured by a microtitration assay using Marc-145 cells in 96-well plates and calculated as 50% tissue culture infective doses (TCID_50_) per milliliter according to the method of Reed and Muench. Each time point was independently repeated three times.

### 2.6. Indirect Immunofluorescence Assay (IFA)

In order to further characterize chimeric or mutant viruses, Marc-145 cells were infected by those viruses and immobilized at 96 hpi; then, they were confirmed with IFA using a monoclonal antibody directed against the PRRSV N protein as described previously [45].

### 2.7. Viral Plaque Morphology Assays

Parental and mutant viruses were infected with Marc-145 cells in 6-well plates. After seven days, the viral plaques were observed after gentian violet staining. In brief, Marc-145 cells were infected with viruses at a dose of MOI = 0.01. After incubating for 1 h, the supernatants were discarded and washed three times with phosphate-buffered saline (PBS). Then, the pre-prepared DMEM medium containing 2% FBS and 1% low-melting agarose was added to the cells. Subsequently, plates were incubated at 37 °C for 5 to 7 days. Eventually, plaque morphology was observed after paraformaldehyde fixation and crystal violet staining. Ten homologous plaques were selected randomly (avoid selecting large plaques formed by fusion of plaques), for which their diameters were measured by a ruler, and finally analyzed by Graphpad Prism 6 (Graphpad, San Diego, CA, USA).

### 2.8. Luciferase Reporter Assay

3′-UTR has several mutant patterns in different PRRSV lineages or branches. In order to detect the transcription activity of 3′-UTR in different mutant patterns, a PRRSV mini-genome system harboring luciferase reporter gene was employed according to a previous study [46]. The PRRSV mini-genome system was a kind gift from Dr. Yan-Dong Tang at the Harbin Veterinary Research Institute. Briefly, 3′-UTR was site-directed mutated according to different lineages and replaced the original 3′-UTR in the PRRSV mini-genome system. Followed by DNA sequencing, the mini-genome system plasmid (0.6 μg) and Renilla luciferase plasmid (0.02 μg) (Promega, Madison, WI, USA) were transfected into Marc-145 cells in 96-well plates. Renilla luciferase activity was used as an internal control for the normalization of luciferase values obtained from cells transfected with the firefly luciferase mini-genome system. At 24 h post-transfection, each well with Marc-145 cells was infected with PRRSV HuN4 at a dose of MOI = 0.1. Twenty-four hours later, the cells were lysed and assayed for firefly luciferase activity using a dual-luciferase reporter assay system (Promega, USA) according to the manufacturer’s instruction.

### 2.9. Statistical Analysis

All experiments were performed at least three independent replicates. All data were analyzed by Graphpad Prism 6. The measured values were expressed as the mean with standard deviation (SD). If no virus was detected by plaque assay, the number representing the LOD (limit of detection) was used. Differences were analyzed for statistical significance using two-tailed unpaired t test for two groups or multiple comparison one-way variance (ANOVA) for more than two groups. Differences were considered statistically significant at a value of *p* < 0.05.

## 3. Results

### 3.1. NSP1, NSP2, NSP7, NSP9 and 3′-UTR Are Closely Related to PRRSV Replication

**Figure 1:**
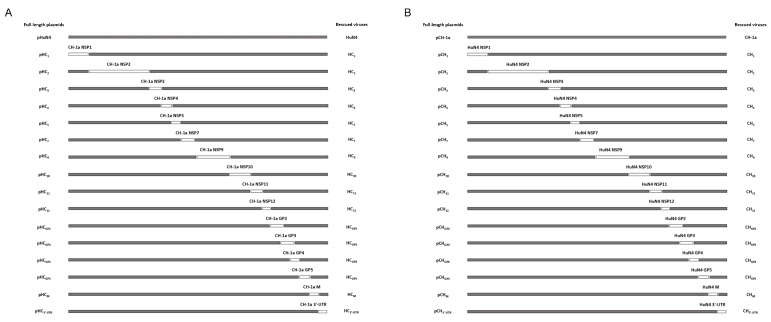
Construction strategy of the full-length chimeric plasmids. (**A**,**B**) Full-length chimeric plasmids were constructed by exchanging the corresponding NSP1, NSP2, NSP3, NSP4, NSP5, NSP7, NSP9, NSP10, NSP11, NSP12, GP2, GP3, GP4, GP5, M and 3′-UTR regions between CH-1a and HuN4 infectious clone. NSP6, NSP8 and N proteins are highly homologous. The boxes represent genomic fragments of parental backbone viruses HuN4 (gray) and CH-1a (white).

**Figure 2:**
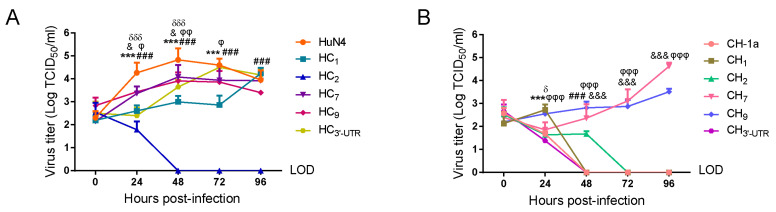
The comparison of replication efficiency between parental and chimeric viruses in PAMs. (**A**,**B**) The viral titers of parental HuN4, CH-1a and their chimeric viruses in PAMs. The parental and mutant viruses infected PAMs at a MOI of 0.01. The data were presented as the mean standard deviation of three independent experiments. LOD: limit of detection. Asterisk (*) indicates a significant difference between HuN4 and CH-1a and HC_1_ and CH_1_, respectively. (***, *p* < 0.001). Pound (#) indicates a significant difference between HuN4 and CH-1a and HC_2_ and CH_2_, respectively. (*###*, *p* < 0.001). And (&) indicates a significant difference between HuN4 and CH-1a and HC_7_ and CH_7_, respectively. (&, *p* < 0.05; &&&, *p* < 0.001). Phi (φ) indicates a significant difference between HuN4 and CH-1a and HC_9_ and CH_9_, respectively. (φ, *p* < 0.05; φφ, *p* < 0.01; φφφ, *p* < 0.001). Delta (δ) indicates a significant difference between HuN4 and CH-1a and HC_3′-UTR_ and CH_3′-UTR_, respectively (*δ*, *p* < 0.05; *δδδ*, *p* < 0.001).

In order to explore the key replication-related viral genes or elements, a series of chimeric viruses by swapping the corresponding genes or elements of HP-PRRSV HuN4 and classical PRRSV CH-1a was constructed based on full-length infectious clones (Figure 1). The replication efficiency of chimeric viruses was compared with their parental viruses in porcine alveolar macrophages (PAMs). The results showed that HC_1_, HC_2_, HC_7_, HC_9_ and HC_3′-UTR_ had significantly lower viral titers than parental HuN4 (Figure 2A). Conversely, CH_7_ and CH_9_ had markedly higher viral titers than the parental CH-1a (Figure 2B). The replication efficiency of other chimeric viruses was not different from their parental viruses (Figure S1).

### 3.2. The Nucleotide at Position 120th in the 3′-UTR Is Critical to Viral Rescue

**Figure 3:**
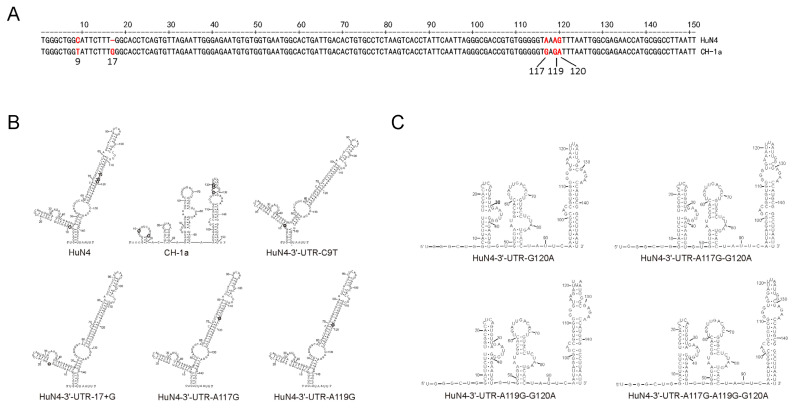
Predicted 3′-UTR secondary structures of parental HuN4 and mutant viruses. (**A**) The alignment of 3′-UTR between HP-PRRSV HuN4 strain and classical PRRSV CH-1a strain. The 3′-UTR differential nucleotides in HuN4 and CH-1a were marked in red. (**B**) The 3′-UTR secondary structures of HuN4, CH-1a, HuN4-3′-UTR-C9T, HuN4-3′-UTR-17+G, HuN4-3′-UTR-A117G and HuN4-3′-UTR-A119G were predicted by mFold method. Different nucleotides between HuN4-3′-UTR and CH-1a-3′-UTR were depicted by hollow round. Mutant nucleotides in the 3′-UTR of HuN4-3′-UTR-C9T, HuN4-3′-UTR-17+G, HuN4-3′-UTR-A117G and HuN4-3′-UTR-A119G were depicted by gray round. (**C**) The 3′-UTR secondary structures of HuN4-3′-UTR-G120A, HuN4-3′-UTR-117+120, HuN4-3′-UTR-119+120 and HuN4-3′-UTR-117+119+120.

The alignment of 3′-UTR between HuN4 and CH-1a showed that there was a 1-nt deletion at position 17th, which was deleted in all HP-PRRSV strains. In addition, there were four mutations (at positions 9, 117, 119, and 120, respectively) in the 3′-UTR of the HP-PRRSV (Figure 3A). In order to investigate the effect of deletion or mutations in the 3′-UTR on viral replication, eight mutant plasmids of pHuN4-3′-UTR-17+G, pHuN4-3′-UTR-C9T, pHuN4-3′-UTR-A117G, pHuN4-3′-UTR-A119G, pHuN4-3′-UTR-G120A, pHuN4-3′-UTR-A117G-G120A, pHuN4-3′-UTR-A119G-G120A and pHuN4-3′-UTR-A117G-A119G-G120A were constructed based on HuN4 infectious clones by replacing the corresponding nucleotide of CH-1a. Only four of them, HuN4-3′-UTR-C9T, HuN4-3′-UTR-17+G, HuN4-3′-UTR-A117G and HuN4-3′-UTR-A119G, were rescued successfully (Figure S2). It may be surprising that a single-point-mutation of 120th or multiple-point-mutation of 120th with other nucleotides were not rescued, indicating the 120th in the 3′-UTR is critical for rescuing HP-PRRSV.

3′-UTR secondary structures of HuN4-3′-UTR-C9T, HuN4-3′-UTR-17+G, HuN4-3′-UTR-A117G and HuN4-3′-UTR-A119G were similar to that of parental HuN4, suggesting that the deletion or mutations at positions 9, 17, 117 and 119 in the 3′-UTR did not change the overall conformation of the 3′-UTR secondary structure (Figure 3B). However, the 3′-UTR secondary structures of HuN4-3′-UTR-G120A, HuN4-3′-UTR-A117G-G120A, HuN4-3′-UTR-A119G-G120A and HuN4-3′-UTR-A117G-A119G-G120A showed obvious differences with that of parental HuN4 (Figure 3C), suggesting the 120th plays a critical role in maintaining the steady conformation of 3′-UTR.

### 3.3. Nucleotides at Position 117th or 119th Significantly Affect Viral Replication

**Figure 4:**
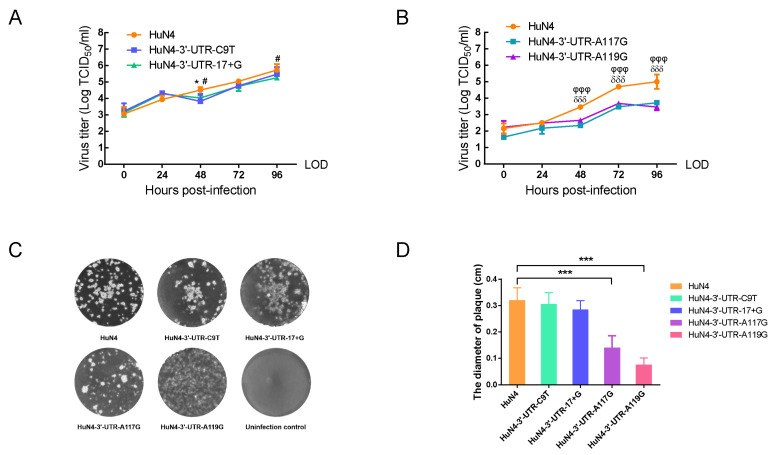
Comparison of replication efficiency of parental and mutant viruses. (**A**) The growth kinetics of parental HuN4 and mutant viruses (HuN4-3′-UTR-C9T and HuN4-3′-UTR-17+G). LOD: limit of detection. Asterisk (*) indicates a significant difference between HuN4 and HuN4-3′-UTR-C9T. (*, *p* < 0.05). Pound (#) indicates a significant difference between HuN4 and HuN4-3′-UTR-17+G (*#*, *p* < 0.05). (**B**) The growth kinetics of parental HuN4 and mutant viruses (HuN4-3′-UTR-A117G and HuN4-3′-UTR-A119G). LOD: limit of detection. Delta (δ) indicates a significant difference between HuN4 and HuN4-3′-UTR-A117G (*δδδ*, *p* < 0.001). Phi (φ) indicates a significant difference between HuN4 and HuN4-3′-UTR-A119G (φφφ, *p* < 0.001). (**C**) Plaque morphology of the parental PRRSV HuN4 and mutant viruses in Marc-145 cells. (**D**) The diameters of viral plaques were measured and analyzed. Asterisk (*) indicates a significant difference between HuN4 and its mutant viruses (***, *p* < 0.001).

After replacement with the nucleotides of CH-1a strain, the replicative level of four mutant viruses was determined and compared with the HuN4 strain in Marc-145 cells. The growth kinetics showed that HuN4-3′-UTR-17+G or HuN4-3′-UTR-C9T displayed a slight decrease (Figure 4A). As for HuN4-3′-UTR-A117G and HuN4-3′-UTR-A119G, their titers were remarkably lower than HuN4 at 48, 72 and 96 hpi (*p* < 0.001) (Figure 4B).

### 3.4. Nucleotides at Position 117th or 119th Significantly Affect Viral Plaques

The average diameters of plaques caused by HuN4-3′-UTR-C9T, HuN4-3′-UTR-17+G, HuN4-3′-UTR-A117G, HuN4-3′-UTR-A119G and HuN4 were 0.305 cm, 0.285 cm, 0.140 cm, 0.075 cm and 0.320 cm, respectively (Figure 4C). The plaques of HuN4-3′-UTR-C9T and HuN4-3′-UTR-17+G were slightly smaller than that of parental HuN4 (Figure 4D). In contrast, the average diameters of plaques caused by HuN4-3′-UTR-A117G were about 33% of the parental HuN4, and HuN4-3′-UTR-A119G was approximately 25% of the parental HuN4 (Figure 4D), suggesting that both mutations at position 117th or 119th closely related to the replication level of HP-PRRSV HuN4 strain.

### 3.5. The 117–120th Motif Was Quite Conservative within Each Lineage

**Figure 5:**
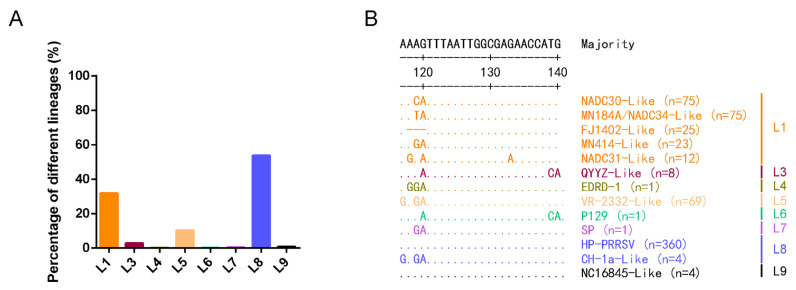
The proportion and mutations in the 3′-UTR motif of different PRRSV lineages. (**A**) The proportion of differential lineage PRRSVs based on all available full-length sequences of PRRSV-2 strains (*n* = 765) in 1991–2019. (**B**) Different mutation patterns of 117–120th of 3′-UTR in different lineage PRRSVs. The representative strain of each lineage, together with the amount of PRRSV strains in corresponding mutation patterns, was listed on the right side of the sequences.

Phylogenetic analysis of all the available full-length PRRSV-2 (*n* = 765) strains from 1992 to 2019 in GenBank showed that those PRRSV-2 were classified into eight lineages (Figure 5A). The results also showed that L8 and L1 were the two most abundant PRRSV-2 strains, both in the world and in China. In the 765 PRRSV-2 strains, the number of strains was as follows: L8 (*n* = 411, 53.73%), L1 (*n* = 243, 31.76%, 208 out of them were detected in 2013-2019), L5 (*n* = 79, 10.33%), L3 (*n* = 21, 2.75%), L9 (*n* = 6, 0.78%), L7 (*n* = 3, 0.39%), L4 (*n* = 1, 0.13%) and L6 (*n* = 1, 0.13%). It also showed that the full-length PRRSV sequences in China contained four different lineages: L8 (*n* = 379, 74.31%), L1 (*n* = 90, 17.65%), L3 (*n* = 21, 4.12%) and L5 (*n* = 20, 3.92%) (Table S1). The alignment of 3′-UTR showed that the 117-120th motif was quite conservative within each lineage (Figure S3). The motif in all L9 PRRSVs was AAAG; L8 was AAAG (in HP-PRRSVs) or GAGA (in classical PRRSVs); L7 was AAGA; L6 was AAAA; L5 was GAGA; L4 was AGGA; and L3 was AAGA or AAAA (Table 2). No L2 full-length sequence has been made available in GenBank yet. In L1 PRRSV, a newly emerging strain that has recently been spreading rapidly, the motif was complex and diverse, such as AACA (in NADC30-like viruses), AGAA (in NADC31-like viruses) and AATA (in MN184A- or NADC34-like viruses) (Figure 5B).

### 3.6. Mutations in the Motif Enhance the Genomic Synthesis of L1 PRRSV

**Figure 6:**
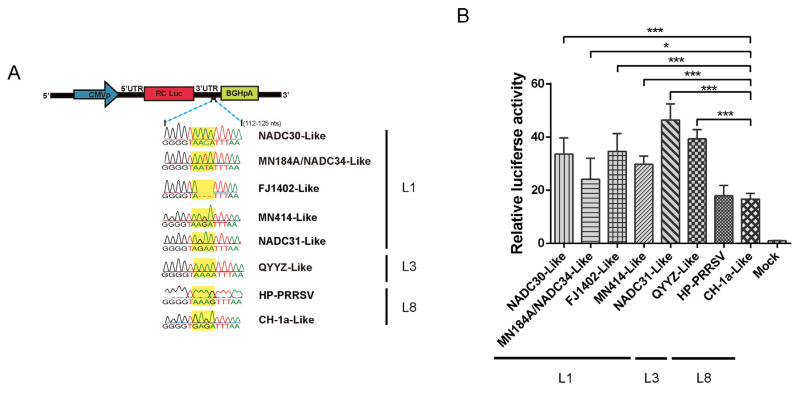
The effects of different 3′-UTR motifs on PRRSV replication. (**A**) Schematic diagram of luciferase plasmids used for Luciferase reporter assay analysis. (**B**) The effects of different 3′-UTR motifs on PRRSV replication. The 3′-UTR mini-genome system mutant plasmids and Renilla luciferase plasmid were co-transfected to Marc-145 cells. After 24 h post-transfection, cells were infected with 0.1 MOI PRRSV HuN4 for another 24 h, and then firefly luciferase activity was measured. Asterisk (*) indicates a significant difference between parental and mutant viruses. (*, *p* < 0.05; ***, *p* < 0.001).

In order to investigate whether the mutations in the motif of L1 were related to viral replication, a PRRSV mini-genome system harboring the 3′-UTR and double luciferase report system was used to evaluate the synthesis level of viral negative genomic RNA, which is essential for genome replication and synthesis. A series of site-directed mutations was performed according to the nucleotides in the motif of different PRRSV lineages (Figure 6A). Firefly luciferase activity was normalized with respect to a co-transfected plasmid encoding Renilla luciferase. The luciferase reporter assay showed that the 3′-UTR of HP-PRRSV HuN4 was 1.2 times than that of classical PRRSV CH-1a, with no significant difference (Figure 6B). All different 3′-UTR of L1 PRRSV exhibited higher replication efficiency than that of classical PRRSV (*p* < 0.05) (Figure 6B), suggesting the RNA synthesis level of L1 PRRSV is higher than that of classical PRRSV.

### 3.7. The 117–120th Motif Are Loacted into a Pseudoknot of 3′-UTR

**Figure 7:**
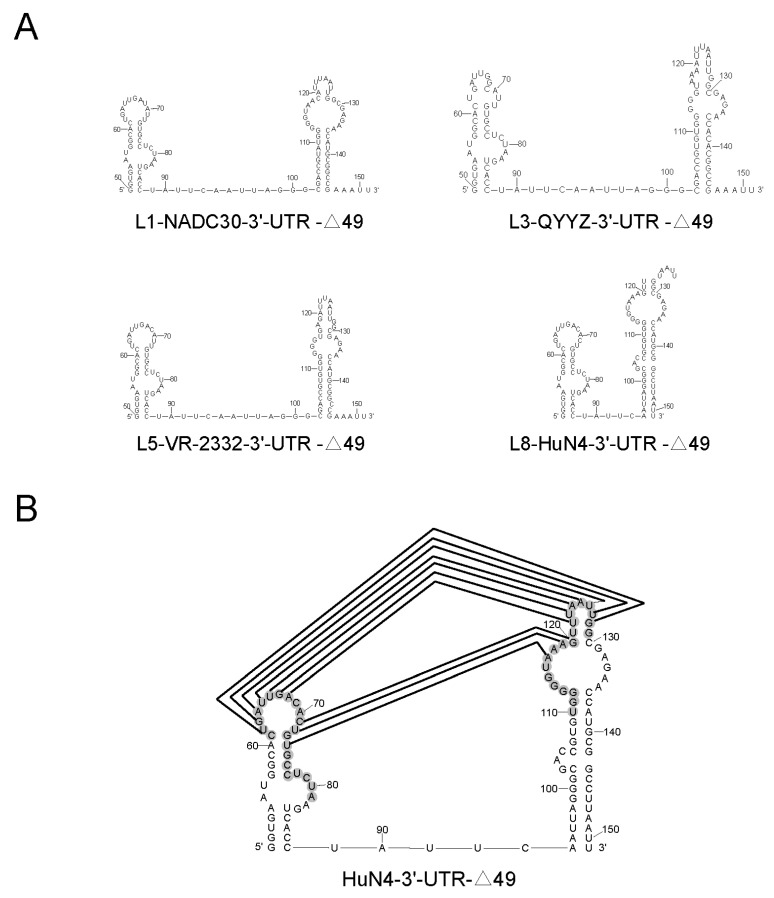
The pseudoknot structure in the 3′-UTR. (**A**) A possible pseudoknot in the 3′-UTR was formed and involved in PRRSV replication after deleting the 49 nucleotides at the 5′-terminal of 3′-UTR. The truncated 3′-UTR secondary structures of L1-NADC30, L3-QYYZ, L5-VR-2332 and L8-HuN4 are shown. (**B**) The nucleotides involving in the formation of pseudoknot are depicted in gray, and base–pairing interaction is depicted by lines. The predicted pseudoknot structure was based on the 3′-UTR sequence of PRRSV HuN4.

3′-UTR secondary structures of representative PRRSVs were predicted in the mFold software (Figure 7A). Meanwhile, the 117–120th motif was located into a pseudoknot (Figure 7B) in the 3′-UTR, according to previous studies [23,43]. The rescued A117G or A119G mutant viruses showed obviously lower replication ability than that of HuN4, while G120A mutant viruses were not successfully rescued, indicating that the 117–120th motif in the 3′-UTR is critical for viral replication or rescue. The mutations at positions 117 and 119 did not change the overall conformation of 3′-UTR secondary structure, but the mutation at position 120 changed the overall secondary structure of 3′-UTR (Figure 3B,C). Nucleotide mutations probably changed the minimum free energy of 3′-UTR secondary structure, which in turn results in alterations in the structure or stability of the pseudoknot.

## 4. Discussion

PRRS has caused severe economic losses to the pig industry since it was reported in 1987. The first PRRSV-2 strain, VR-2332 (belonging to L5), was isolated in the US in 1992. In China, the first PRRSV-2 strain, CH-1a (classical PRRSV, belonging to L8), was isolated in 1996. With the gradual evolution of PRRSV, HP-PPRSV (belonging to L8) emerged and spread rapidly, and they quickly became the dominant epidemic strains [5]. HP-PRRSV was well documented, and it has higher replicative ability than classical PRRSV [32,33], which helps in understanding why HP-PRRSV spread rapidly. However, the key replication-regulated genes or elements still have not been elucidated.

Our study showed that NSP1, NSP2, NSP7, NSP9 and 3′-UTR are closely related to the replication efficiency of HP-PRRSV. Moreover, previous studies demonstrate that NSP1 has an effect on the synthesis of TNF-α and IFN-β [47,48]; NSP2, NSP3 and NSP5 interact with each other and participate in RTC formation and viral replication [19,49]; NSP7 interacts with NSP9, and NSP9 contributes to the replication ability and pathogenicity of PRRSV [33,50]. Meanwhile, the NSP2, NSP3, NSP5, NSP9 and NSP10 were reported to assemble in viral replication and transcription complex (RTC); then, RTC interacts with 3′-UTR, which initiates the transcription of sgmRNA and the replication of viral genome [19,20]. However, as a critical element of RTC, the function and key regions or sites of 3′-UTR in viral replication are still unclear. Therefore, a follow-up study on 3′-UTR was performed. By swapping the corresponding genes between CH-1a and HuN4, we demonstrated that the 3′-UTR contributes to the enhanced replicative ability of HP-PRRSV. Due to the low replication ability of CH-1a, the chimeric or mutant viruses based on the backbone of CH-1a were not replicated well in Marc-145 or PAM cells. Thus, the site-direct mutations of 3′-UTR were based on the HuN4 strain. Growth kinetics and plaque morphology assays show that the nucleotides at positions 117th and 119th in the 3′-UTR significantly affect viral replication or plaque sizes, suggesting that two nucleotides are critical sites for viral replication. Interestingly, the 120th nucleotide in the 3′-UTR is important for viral rescue, which may be because the 120th nucleotide and those nearby are involved in the formation of a senior RNA structure. Therefore, the 120th and its nearby nucleotides (117th to 119th) consist of a novel motif, 117–120th, which is a key motif related to PRRSV replication.

Alignment of all available entire sequences of PRRSV-2 in 1992–2019 revealed that the 117–120th nucleotides in the 3′-UTR were conservative in individual lineages. For each lineage, there were more or less mutations in the motif when compared with the prototype PRRSV-2, VR-2332 strain. The mutations contribute to the higher replication efficiency of HP-PRRSV HuN4 compared with the CH-1a strain. A highly efficient replication in host cells is one of the important features of dominant epidemic strain. There are many factors that result in HP-PRRSV becoming the dominant epidemic strain, such as increased viral tropism, high level of viral replication, moderate pathogenicity and so on [33,50,51].

In China, L1 PRRSV emerged in 2013, then it spread quickly in main pig-raising provinces since 2015, and L1 PRRSVs mainly include NADC30-like PRRSV and NADC34-like PRRSV [6,31]. At present, HP-PRRSV and L1 PRRSVs are the dominant epidemic strains, and the proportion of L1 PRRSVs is higher than that of HP-PRRSV in some provinces [5,6,30]. There is a trend that the L1 PRRSVs surpass HP-PRRSV to become the most dominant epidemic strain in the next few years. In the present study, we found that mutations in the motif of 3′-UTR enhanced the replication of HP-PRRSV or L1 PRRSVs. At present, it is suitable for the most prevalent PRRSV lineages (L1/L3/L4/L5/L6/L7/L8/L9, 96.20%), except the FJ1402-like strain with deletion at positions 118–120th (L1, 3.80%). FJ1402-like strains with deletion at positions 118–120th in 3′-UTR have increased in recent years, and the detailed relationship between deletion and viral replication remains to be further explored.

Pseudoknot in the 3′-UTR of PRRSV can serve as pathogen-associated molecular patterns (PAMPs) and bind to the host cell pattern recognition receptors (PRRs) TLR3 and RIG-I to activate innate immune signaling and produced IFNs [43]. A previous study revealed a hairpin structure in the ORF7 together with the 3′-UTR form a kissing structure, which is essential for PRRSV replication [42]. In Mengovirus, three stem-loop (SL) are formed in its 3′-UTR, in which SL1 is a non-essential region for viral replication, and the absence of SL2 reduces the viral replication capability, and SL3 is necessary for viral replication [52]. In Dengue virus, SLs (A2 and A3) in the D2 region of 3′-UTR are essential for viral replication [53]. Consequently, we speculate that the 117-120th motif plays an important role in PRRSV replication. A pseudoknot is formed in the 3′-UTR according to the previous RNA structure analysis method [23], and the 117–120th motif was located in the pseudoknot.

## 5. Conclusions

In summary, our study has revealed that (i) NSP1, NSP2, NSP7, NSP9 and 3′-UTR are closely related to replication efficiency of PRRSV-2; (ii) the nucleotides at positions 117th and 119th in the 3′-UTR are critical for viral replication; (iii) the 117–120th is an important motif in the 3′-UTR of PRRSV-2, and mutations in the motif contribute to the higher replication level of HP-PRRSV or L1 PRRSVs. Our findings discovered the key motif or nucleotide involved in viral replication and would help to understand the mechanism of viral replication. The motif is potentially related to the formation of dominant epidemic of HP-PRRSV strains or L1 PRRSVs, which will require further investigation.

## Figures and Tables

**Table 1:** Primers used in this study.

Primers	Sequences (5′-3′)	Application
F1	GCGACGCGTTTGTCGTCCGGCGTC	Mutant clone
R2	GCCTTAATTAAGGCCGCATGGTT	
C9T-R1	GCCAAAGAATACCAGCCCATC	HuN4-3′-UTR-C9T clone
C9T-F2	GATGGGCTGGTATTCTTTGGC	
17+G-R1	ACACTGAGGTGCCCAAAGAAT	HuN4-3′-UTR-17+G clone
17+G-F2	ATTCTTTGGGCACCTCAGTGT	
A117G-R	GCCTTAATTAAGGCCGCATGGTTCTCGCCAATTAAACTTCACCCCCACAC	HuN4-3′-UTR-A117G clone
A119G-R	GCCTTAATTAAGGCCGCATGGTTCTCGCCAATTAAACCTTACCCCCACA	HuN4-3′-UTR-A119G clone
G120A-R	GCCTTAATTAAGGCCGCATGGTTCTCGCCAATTAAATTTTACCCCC	HuN4-3′-UTR-G120A clone
A117G-G120A-R	GCCTTAATTAAGGCCGCATGGTTCTCGCCAATTAAATTTCACCCCCACAC	HuN4-3′-UTR-A117G-G120A clone
A119G-G120A-R	GCCTTAATTAAGGCCGCATGGTTCTCGCCAATTAAATCTTACCCCCACAC	HuN4-3′-UTR-A119G-G120A clone
A117G-A119G-G120A-R	GCCTTAATTAAGGCCGCATGGTTCTCGCCAATTAAATCTCACCCCCACAC	HuN4-3′-UTR-A117G-A119G-G120A clone

**Table 2:** Mutations of the 117–120th motif in different PRRSV lineages. L1 includes five mutation patterns: AATA (represented by MN184A), AACA (represented by NADC30), AAGA, AGAA and deletion at position 118–120th. L3 includes two mutation patterns: AAAA and AAGA. L8 includes five dominant mutation patterns: AAAG (represented by HuN4), AAAA, TAAG, AGAG and GAGA (represented by CH-1a).

Lineages	Representative Strains	The 117–120th Motif in the 3′-UTR				
L1	MN184A/NADC30	AATA (75, 30.86%)	AACA (75, 30.86%)	Deletion (25, 10.29%)	AAGA (23, 9.47%)	AGAA (12, 4.94%)
L3	QYYZ	AAAA (8, 38.10%)	AAGA (6, 28.57%)			
L4	EDRD-1	AGGA (1, 100%)				
L5	VR-2332	GAGA (69, 87.34%)				
L6	P129	AAAA (1, 100%)				
L7	SP	AAGA (3, 100%)				
L8	HuN4/CH-1a	AAAG (360, 87.59%)	AAAA (18, 4.38%)	TAAG (7, 1.70%)	AGAG (5, 1.22%)	GAGA (4, 0.79%)
L9	NC16845	AAAG (4, 66.67%)				

## Data Availability

All available data are presented in the article.

## Supplementary Materials

The following supporting information can be downloaded at: https://www.mdpi.com/article/10.3390/v14020166/s1, Figure S1: The comparison of replication efficiency between parental and chimeric viruses in PAMs; Figure S2: Identification of mutant viruses; Figure S3: The 3′-UTR alignment of 765 PRRSV-2 strains. Table S1: Information of 765 NA-type PRRSVs in 1992–2019.

## References

[1] Neumann E.J., Kliebenstein J.B., Johnson C.D., Mabry J.W., Bush E.J., Seitzinger A.H., Green A.L., Zimmerman J.J. (2005). Assessment of the economic impact of porcine reproductive and respiratory syndrome on swine production in the United States. J. Am. Vet. Med. Assoc..

[2] Albina E. (1997). Epidemiology of porcine reproductive and respiratory syndrome (PRRS): An overview. Vet. Microbiol..

[3] Wensvoort G., Terpstra C., Pol J.M., Ter Laak E.A., Bloemraad M., de Kluyver E.P., Kragten C., van Buiten L., den Besten A., Wagenaar F. (1991). Mystery swine disease in the Netherlands: The isolation of Lelystad virus. Vet. Q..

[4] Wensvoort G., de Kluyver E.P., Pol J.M., Wagenaar F., Moormann R.J., Hulst M.M., Bloemraad R., den Besten A., Zetstra T., Terpstra C. (1992). Lelystad virus, the cause of porcine epidemic abortion and respiratory syndrome: A review of mystery swine disease research at Lelystad. Vet. Microbiol..

[5] Gao J.C., Xiong J.Y., Ye C., Chang X.B., Guo J.C., Jiang C.G., Zhang G.H., Tian Z.J., Cai X.H., Tong G.Z. (2017). Genotypic and geographical distribution of porcine reproductive and respiratory syndrome viruses in mainland China in 1996–2016. Vet. Microbiol..

[6] Yu F., Yan Y., Shi M., Liu H.Z., Zhang H.L., Yang Y.B., Huang X.Y., Gauger P.C., Zhang J., Zhang Y.H. (2020). Phylogenetics, genomic recombination, and NSP2 polymorphic patterns of porcine reproductive and respiratory syndrome virus in China and the United States in 2014–2018. J. Virol..

[7] Conzelmann K.K., Visser N., Van Woensel P., Thiel H.J. (1993). Molecular characterization of porcine reproductive and respiratory syndrome virus, a member of the arterivirus group. Virology.

[8] Wu W.H., Fang Y., Farwell R., Steffen-Bien M., Rowland R.R., Christopher-Hennings J., Nelson E.A. (2001). A 10-kDa structural protein of porcine reproductive and respiratory syndrome virus encoded by ORF2b. Virology.

[9] Johnson C.R., Griggs T.F., Gnanandarajah J., Murtaugh M.P. (2011). Novel structural protein in porcine reproductive and respiratory syndrome virus encoded by an alternative ORF5 present in all arteriviruses. J. Gen. Virol..

[10] Snijder E.J., van Tol H., Roos N., Pedersen K.W. (2001). Non-structural proteins 2 and 3 interact to modify host cell membranes during the formation of the arterivirus replication complex. J. Gen. Virol..

[11] Fang Y., Kim D.Y., Ropp S., Steen P., Christopher-Hennings J., Nelson E.A., Rowland R.R. (2004). Heterogeneity in Nsp2 of European-like porcine reproductive and respiratory syndrome viruses isolated in the United States. Virus Res..

[12] den Boon J.A., Faaberg K.S., Meulenberg J.J., Wassenaar A.L., Plagemann P.G., Gorbalenya A.E., Snijder E.J. (1995). Processing and evolution of the N-terminal region of the arterivirus replicase ORF1a protein: Identification of two papainlike cysteine proteases. J. Virol..

[13] van Dinten L.C., Wassenaar A.L., Gorbalenya A.E., Spaan W.J., Snijder E.J. (1996). Processing of the equine arteritis virus replicase ORF1b protein: Identification of cleavage products containing the putative viral polymerase and helicase domains. J. Virol..

[14] Fang Y., Treffers E.E., Li Y., Tas A., Sun Z., van der Meer Y., de Ru A.H., van Veelen P.A., Atkins J.F., Snijder E.J. (2012). Efficient −2 frameshifting by mammalian ribosomes to synthesize an additional arterivirus protein. Proc. Natl. Acad. Sci. USA.

[15] Snijder E.J., Wassenaar A.L., Spaan W.J. (1994). Proteolytic processing of the replicase ORF1a protein of equine arteritis virus. J. Virol..

[16] Wang T.Y., Fang Q.Q., Cong F., Liu Y.G., Wang H.M., Zhang H.L., Tian Z.J., Tang Y.D., Cai X.H. (2019). The Nsp12-coding region of type 2 PRRSV is required for viral subgenomic mRNA synthesis. Emerg. Microbes Infec..

[17] Wassenaar A.L., Spaan W.J., Gorbalenya A.E., Snijder E.J. (1997). Alternative proteolytic processing of the arterivirus replicase ORF1a polyprotein: Evidence that NSP2 acts as a cofactor for the NSP4 serine protease. J. Virol..

[18] Wootton S., Yoo D., Rogan D. (2000). Full-length sequence of a Canadian porcine reproductive and respiratory syndrome virus (PRRSV) isolate. Arch. Virol..

[19] Nan H., Lan J., Tian M., Dong S., Tian J., Liu L., Xu X., Chen H. (2018). The network of interactions among porcine reproductive and respiratory syndrome virus non-structural proteins. Front. Microbiol..

[20] Fang Y., Snijder E.J. (2010). The PRRSV replicase: Exploring the multifunctionality of an intriguing set of nonstructural proteins. Virus Res..

[21] Zhu L., Bao L., Zhang X., Xia X., Sun H. (2015). Inhibition of porcine reproductive and respiratory syndrome virus replication with exosome-transferred artificial microRNA targeting the 3′ untranslated region. J. Virol. Methods.

[22] Goebel S.J., Hsue B., Dombrowski T.F., Masters P.S. (2004). Characterization of the RNA components of a putative molecular switch in the 3′ untranslated region of the murine coronavirus genome. J. Virol..

[23] Beerens N., Snijder E.J. (2007). An RNA pseudoknot in the 3′ end of the arterivirus genome has a critical role in regulating viral RNA synthesis. J. Virol..

[24] Wang D., Ge X., Chen D., Li J., Cai Y., Deng J., Zhou L., Guo X., Han J., Yang H. (2018). The S gene is necessary but not sufficient for the virulence of porcine epidemic diarrhea virus novel variant strain BJ2011C. J. Virol..

[25] Filomatori C.V., Bardossy E.S., Merwaiss F., Suzuki Y., Henrion A., Saleh M.C., Alvarez D.E. (2019). RNA recombination at Chikungunya virus 3′UTR as an evolutionary mechanism that provides adaptability. PLoS Pathog..

[26] Nelsen C.J., Murtaugh M.P., Faaberg K.S. (1999). Porcine reproductive and respiratory syndrome virus comparison: Divergent evolution on two continents. J. Virol..

[27] Guo B.Q., Chen Z.S., Liu W.X. (1996). Porcine reproductive and respiratory syndrome virus was isolated from abortive fetus of suspected PRRS. Chin. J. Anim. Poultry Infect. Dis..

[28] An T.Q., Tian Z.J., Xiao Y., Li R., Peng J.M., Wei T.C., Zhang Y., Zhou Y.J., Tong G.Z. (2010). Origin of highly pathogenic porcine reproductive and respiratory syndrome virus, China. Emerg. Infect. Dis..

[29] An T.Q., Tian Z.J., Leng C.L., Peng J.M., Tong G.Z. (2011). Highly pathogenic porcine reproductive and respiratory syndrome virus, Asia. Emerg. Infect. Dis..

[30] Han J., Zhou L., Ge X., Guo X., Yang H. (2017). Pathogenesis and control of the Chinese highly pathogenic porcine reproductive and respiratory syndrome virus. Vet. Microbiol..

[31] Zhao K., Ye C., Chang X.B., Jiang C.G., Wang S.J., Cai X.H., Tong G.Z., Tian Z.J., Shi M., An T.Q. (2015). Importation and recombination are responsible for the latest emergence of highly pathogenic porcine reproductive and respiratory syndrome virus in China. J. Virol..

[32] Dong J., Wang G., Liu Y., Shi W., Wu J., Wen H., Wang S., Tian Z., Cai X. (2016). Quantitative estimation of the replication kinetics of genotype 2 PRRSV strains with different levels of virulence in vitro. J. Virol. Methods.

[33] Li Y., Zhou L., Zhang J., Ge X., Zhou R., Zheng H., Geng G., Guo X., Yang H. (2014). Nsp9 and Nsp10 contribute to the fatal virulence of highly pathogenic porcine reproductive and respiratory syndrome virus emerging in China. PLoS Pathog..

[34] Kumar S., Stecher G., Tamura K. (2016). MEGA7: Molecular evolutionary genetics analysis version 7.0 for bigger datasets. Mol. Biol. Evol..

[35] Shi M., Lam T.T., Hon C.C., Murtaugh M.P., Davies P.R., Hui R.K., Li J., Wong L.T., Yip C.W., Jiang J.W. (2010). Phylogeny-based evolutionary, demographical, and geographical dissection of North American type 2 porcine reproductive and respiratory syndrome viruses. J. Virol..

[36] An T.Q., Tian Z.J., Zhou Y.J., Xiao Y., Peng J.M., Chen J., Jiang Y.F., Hao X.F., Tong G.Z. (2011). Comparative genomic analysis of five pairs of virulent parental/attenuated vaccine strains of PRRSV. Vet. Microbiol..

[37] Zuker M. (2003). Mfold web server for nucleic acid folding and hybridization prediction. Nucleic Acids Res..

[38] De Rijk P., Wuyts J., De Wachter R. (2003). RnaViz 2: An improved representation of RNA secondary structure. Bioinformatics.

[39] Olsthoorn R.C., Mertens S., Brederode F.T., Bol J.F. (1999). A conformational switch at the 3′ end of a plant virus RNA regulates viral replication. Embo.J..

[40] Williams G.D., Chang R.Y., Brian D.A. (1999). A phylogenetically conserved hairpin-type 3′ untranslated region pseudoknot functions in coronavirus RNA replication. J. Virol..

[41] Hsue B., Hartshorne T., Masters P.S. (2000). Characterization of an essential RNA secondary structure in the 3′ untranslated region of the murine coronavirus genome. J. Virol..

[42] Verheije M.H., Olsthoorn R.C., Kroese M.V., Rottier P.J., Meulenberg J.J. (2002). Kissing interaction between 3′ noncoding and coding sequences is essential for porcine arterivirus RNA replication. J. Virol..

[43] Xie S., Chen X.X., Qiao S., Li R., Sun Y., Xia S., Wang L.J., Luo X., Deng R., Zhou E.M. (2018). Identification of the RNA pseudoknot within the 3′ end of the porcine reproductive and respiratory syndrome virus genome as a pathogen-associated molecular pattern to activate antiviral signaling via RIG-I and toll-like receptor 3. J. Virol..

[44] Reed L.J., Muench H. (1938). A simple method of estimating fifty percent endpoints. Am. J. Hyg..

[45] Yin Y., Liu C., Liu P., Yao H., Wei Z., Lu J., Tong G., Gao F., Yuan S. (2013). Conserved nucleotides in the terminus of the 3′ UTR region are important for the replication and infectivity of porcine reproductive and respiratory syndrome virus. Arch. Virol..

[46] Tang Y.D., Fang Q.Q., Liu J.T., Wang T.Y., Wang Y., Tao Y., Liu Y.G., Cai X.H. (2016). Open reading frames 1a and 1b of the porcine reproductive and respiratory syndrome virus (PRRSV) collaboratively initiate viral minus-strand RNA synthesis. Biochem. Biophys. Res. Commun..

[47] Chen Z., Lawson S., Sun Z., Zhou X., Guan X., Christopher-Hennings J., Nelson E.A., Fang Y. (2010). Identification of two auto-cleavage products of nonstructural protein 1 (nsp1) in porcine reproductive and respiratory syndrome virus infected cells: Nsp1 function as interferon antagonist. Virology.

[48] Subramaniam S., Kwon B., Beura L.K., Kuszynski C.A., Pattnaik A.K., Osorio F.A. (2010). Porcine reproductive and respiratory syndrome virus non-structural protein 1 suppresses tumor necrosis factor-α promoter activation by inhibiting NF-kB and Sp1. Virology.

[49] Chen J., Xu X., Tao H., Li Y., Nan H., Wang Y., Tian M., Chen H. (2017). Structural analysis of porcine reproductive and respiratory syndrome virus non-structural protein 7α (NSP7α) and identification of its interaction with NSP9. Front. Microbiol..

[50] Zhao K., Gao J.C., Xiong J.Y., Guo J.C., Yang Y.B., Jiang C.G., Tang Y.D., Tian Z.J., Cai X.H., Tong G.Z. (2018). Two residues in NSP9 contribute to the enhanced replication and pathogenicity of highly pathogenic porcine reproductive and respiratory syndrome virus. J. Virol..

[51] Zhang H.L., Tang Y.D., Liu C.X., Xiang L.R., Zhang W.L., Leng C.L., Wang Q., An T.Q., Peng J.M., Tian Z.J. (2018). Adaptions of field PRRSVs in Marc-145 cells were determined by variations in the minor envelope proteins GP2a-GP3. Vet. Microbiol..

[52] Duque H., Palmenberg A.C. (2001). Phenotypic characterization of three phylogenetically conserved stem-loop motifs in the Mengovirus 3′ untranslated region. J. Virol..

[53] Alvarez D.E., Lodeiro M.F., Luduena S.J., Pietrasanta L.I., Gamarnik A.V. (2005). Long-range RNA-RNA interactions circularize the dengue virus genome. J. Virol..

