# Supplementary material for: A Novel Motif in the 3′-UTR of PRRSV-2 Is Critical for Viral Multiplication and Contributes to Enhanced Replication Ability of Highly Pathogenic or L1 PRRSV
Source: Viruses. 2022 Jan 18;14(2):166. doi: 10.3390/v14020166 (PMC8875199; doi:10.3390/v14020166)
Supplement: Supplementary file 1 [file viruses-14-00166-s001.zip › PDF/Fig S3. The 3'-UTR alignment of 765 PRRSV-2 strains.pdf]

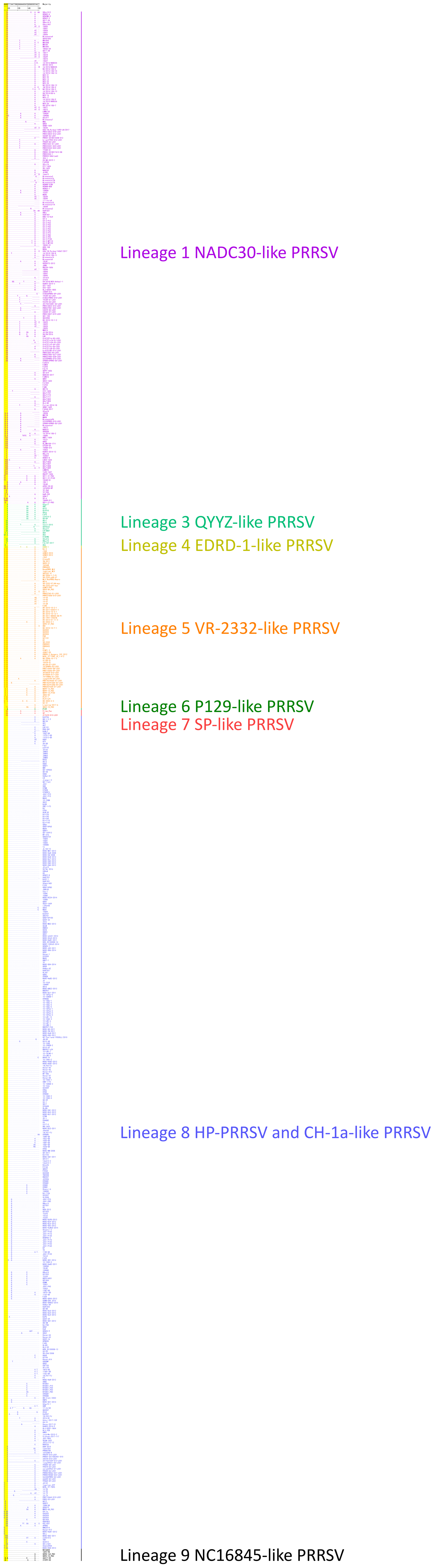
Lineage 1 NADC30-like PRRSV
Lineage 3 QYYZ-like PRRSV
Lineage 4 EDRD-1-like PRRSV
Lineage 5 VR-2332-like PRRSV
Lineage 6 P129-like PRRSV
Lineage 7 SP-like PRRSV
Lineage 8 HP-PRRSV and CH-1a-like PRRSV
Lineage 9 NC16845-like PRRSV
