# Supplementary material for: A Novel Motif in the 3′-UTR of PRRSV-2 Is Critical for Viral Multiplication and Contributes to Enhanced Replication Ability of Highly Pathogenic or L1 PRRSV
Source: Viruses. 2022 Jan 18;14(2):166. doi: 10.3390/v14020166 (PMC8875199; doi:10.3390/v14020166)
Supplement: Supplementary file 1 [file viruses-14-00166-s001.zip › Supplementary Material.pdf]

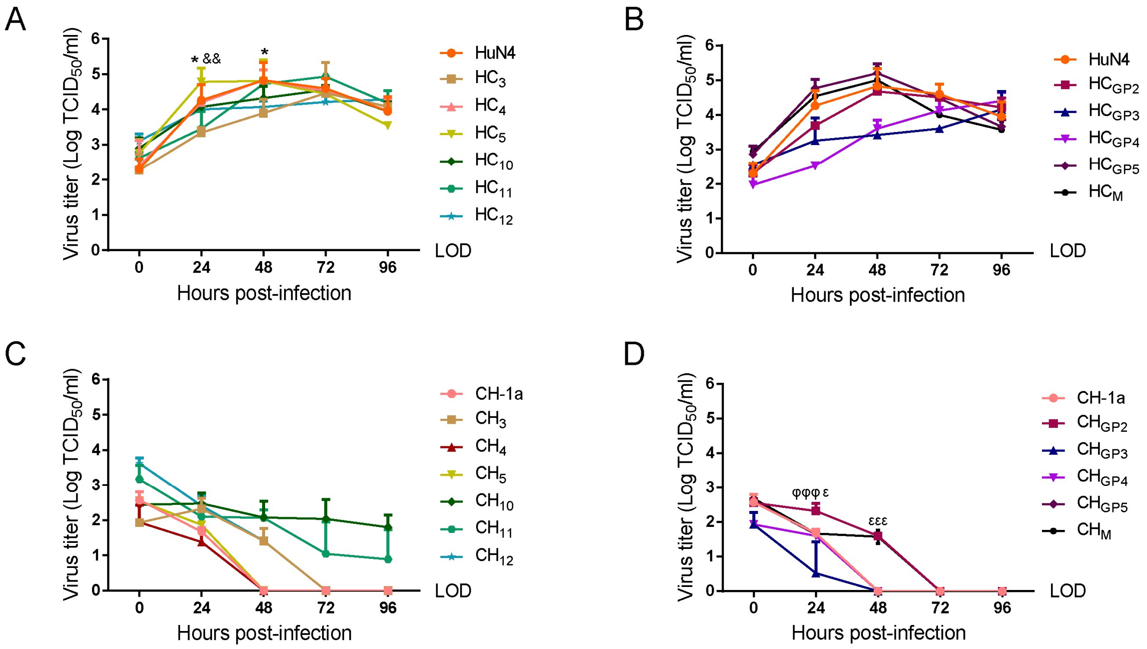

Figure S1. The comparison of replication efficiency between parental and chimeric viruses in PAMs. (A, B, C, D) The growth kinetics between parental HuN4, CH-1a and their chimeric viruses in PAMs. The parental and mutant viruses are infected with PAMs at a multiplicity of infection (MOI) of 0.01. The cell supernatants are harvested at 0, 24, 48, 72 and 96 hours post-infection (hpi). Virus titers from 24 h to 96 h are determined by microtitration infectivity assays, respectively. The data are presented as the mean standard deviation (shown by error bars) from three independent experiments. LOD: limit of detection. Asterisk (*) indicates a significant difference between HuN4, CH-1a and HC_3, CH_3, respectively. (*, $p < 0.05$). And (&) indicates a significant difference between HuN4, CH-1a and HC_{11}, CH_{11}, respectively. (&&, $p < 0.01$). Epsilon (ε) indicates a significant difference between HuN4, CH-1a and HC_{GP2}, CH_{GP2}, respectively. (ε, $p < 0.05$; εεε, $p < 0.001$). Phi (φ) indicates a significant difference between HuN4, CH-1a and HC_{GP4}, CH_{GP4}, respectively. (φφφ, $p < 0.001$).

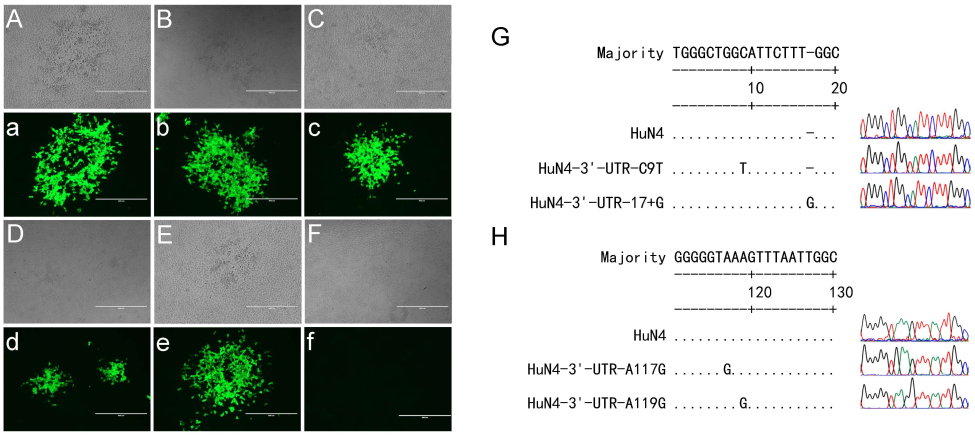

Figure S2. Identification of mutant viruses. (A, B, C, D, E, F) The cytopathic effect of HuN4, HuN4-3'-UTR-C9T, HuN4-3'-UTR-19+G, HuN4-3'-UTR-A117G, HuN4-3'-UTR-A119G and negative control under microscope. The parental and mutant viruses are infected with Marc-145 cells at 80 % confluence and observed under light microscope after 4 or 5 days. (a, b, c, d, e, f) Identification of HuN4, HuN4-3'-UTR-C9T, HuN4-3'-UTR-19+G, HuN4-3'-UTR-A117G, HuN4-3'-UTR-A119G and negative control by IFA. The parental and mutant viruses are infected with Marc-145 cells at 80 % confluence and observed under inverted fluorescence microscope. (G, H) The viruses of HuN4, HuN4-3'-UTR-C9T, HuN4-3'-UTR-19+G, HuN4-3'-UTR-A117G, HuN4-3'-UTR-A119G are sequenced and analyzed using MegAlign and Chromas.

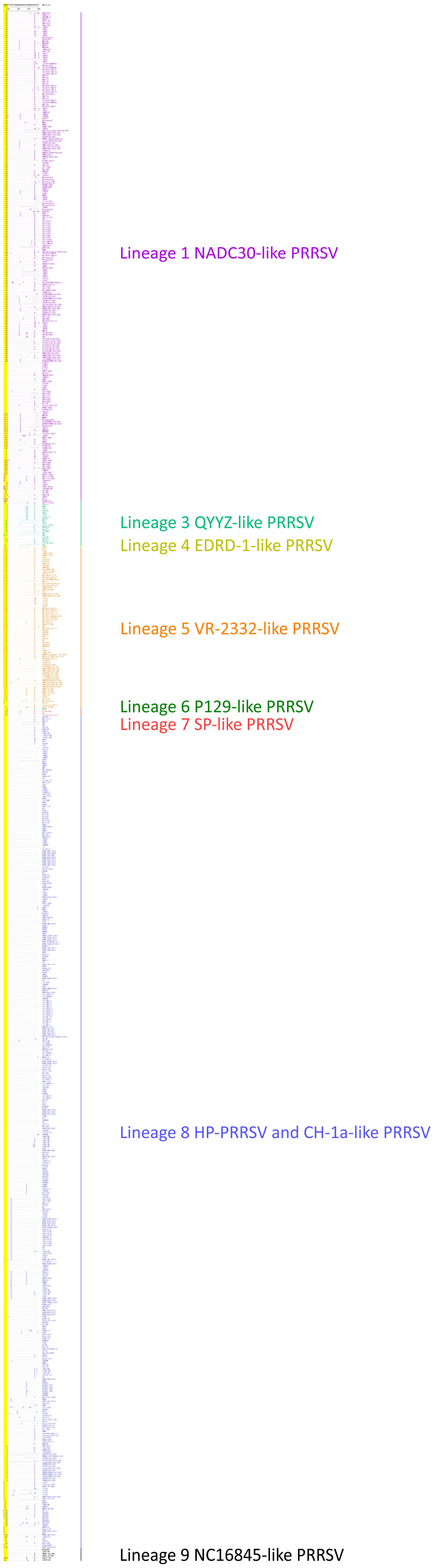
Lineage 1 NADC30-like PRRSV
Lineage 3 QYYZ-like PRRSV
Lineage 4 EDRD-1-like PRRSV
Lineage 5 VR-2332-like PRRSV
Lineage 6 P129-like PRRSV
Lineage 7 SP-like PRRSV
Lineage 8 HP-PRRSV and CH-1a-like PRRSV
Lineage 9 NC16845-like PRRSV

Figure S3. The 3'-UTR alignment of 765 PRRSV-2 strains. The purple, cyan, buff, orange, light green, jacinth, blue and black represent the L1-NADC30-like PRRSV, L3-QYYZ-like PRRSV, L4-EDRD-1-like PRRSV, L5-VR-2332-like PRRSV, L6-P129-like PRRSV, L7-SP-like PRRSV, L8-HP-PRRSV and CH-1a-like PRRSV, and L9-NC16845-like PRRSV strains, respectively. The four conserved nucleotides are highlighted in yellow.
