# Supplementary material for: A Novel Motif in the 3′-UTR of PRRSV-2 Is Critical for Viral Multiplication and Contributes to Enhanced Replication Ability of Highly Pathogenic or L1 PRRSV
Source: Viruses. 2022 Jan 18;14(2):166. doi: 10.3390/v14020166 (PMC8875199; doi:10.3390/v14020166)
Supplement: Supplementary file 1 [file viruses-14-00166-s001.zip › Table S1. Information of 765 NA-type PRRSVs in 1992-2019.pdf]

Supplementary table S1. Information of 765 NA-type PRRSVs in 1992-2019

Accession number	Strain name	Year	Country	Lineage
KY348849	18565-01	2001	USA	L1
DQ473474	LMY	2002	South Korea	L1
KY348848	18066-04	2004	USA	L1
JN654459	NADC30	2008	USA	L1
JN660150	NADC31	2008	USA	L1
KC862579	DK-2010-10-1-2	2010	Denmark	L1
KP283404	Minnesota16	2011	USA	L1
KU523367	WUH6	2011	China	L1
KU131566	SD11-21-P84	2011	USA	L1
KU131568	SD11-21-P100	2011	USA	L1
KU131557	SD11-21-P83	2011	USA	L1
KP283409	Minnesota7	2012	USA	L1
KP283411	Minnesota4	2012	USA	L1
KP283401	MN6	2012	USA	L1
KP283403	Minnesota17A	2012	USA	L1
KP283410	Minnesota5	2012	USA	L1
KP283405	Minnesota15	2012	USA	L1
KP283412	Minnesota3	2012	USA	L1
KP283416	Illinois8	2012	USA	L1
KP283407	Minnesota11A	2012	USA	L1
KP283406	Minnesota14	2012	USA	L1
KJ143621	HENAN-HEB	2012	China	L1
KP283415	Iowa12	2012	USA	L1
KP283413	Minnesota2	2012	USA	L1
KP283399	MN11B	2012	USA	L1
KP283400	MN9B	2012	USA	L1
KP283408	Minnesota9A	2012	USA	L1
KP283414	Minnesota1	2012	USA	L1
KP283402	Minnesota17B	2013	USA	L1
KF555450	CA-2	2013	South Korea	L1
KF555451	KNU-12-KJ4	2013	South Korea	L1
KF611905	HENAN-XINX	2013	China	L1
KR534893	OH28372-2013	2013	USA	L1
KR706343	JL580	2013	China	L1
KP860909	FJZ03	2013	China	L1
KP860911	FJW05	2013	China	L1
MF326988	IA/2013/ISU-1	2013	USA	L1
MF326985	IA/2014/NADC34	2014	USA	L1
KT257966	ISU10	2014	USA	L1
KT257959	ISU01	2014	USA	L1
KP861625	CHsx1401	2014	China	L1
MF326991	NC/2014/ISU-4	2014	USA	L1

MF326993	OH/2014/ISU-6	2014	USA	L1
MF326994	OH/2014/ISU-7	2014	USA	L1
MF326992	IN/2014/ISU-5	2014	USA	L1
KT257962	ISU04	2014	USA	L1
KT257965	ISU07	2014	USA	L1
KT257974	ISU27	2014	USA	L1
KT257964	ISU06	2014	USA	L1
KT257963	ISU05	2014	USA	L1
KT257967	ISU17	2014	USA	L1
KT257968	ISU18	2014	USA	L1
KT257976	ISU29	2014	USA	L1
KT257977	ISU30	2014	USA	L1
KT257990	ISU71	2014	USA	L1
KT257991	ISU72	2014	USA	L1
MF326990	NC/2014/ISU-3	2014	USA	L1
KT257987	ISU68	2014	USA	L1
KT258001	ISU94	2014	USA	L1
KT258003	ISU97	2014	USA	L1
KT258004	ISU95	2014	USA	L1
KT257954	21675	2014	USA	L1
KT257955	101416	2014	USA	L1
KU950372	HENXX-1	2014	China	L1
KY373214	JSWA	2014	China	L1
KT257981	ISU39	2014	USA	L1
KT257982	ISU40	2014	USA	L1
KT258002	ISU96	2014	USA	L1
MF766471	HeN1401	2014	China	L1
MF326995	IA/2014/ISU-8	2014	USA	L1
MN046226	HeNXX-2014-3	2014	China	L1
KT581982	MN414	2014	USA	L1
KT257973	ISU25	2014	USA	L1
KT257970	ISU22	2014	USA	L1
KT257972	ISU24	2014	USA	L1
KT257971	ISU23	2014	USA	L1
KX169191	FJ1402	2014	China	L1
KP860910	FJY04	2014	China	L1
KY412887	FJL15	2014	China	L1
KY412888	FJM4	2014	China	L1
MN046225	Fujian-2014-18	2014	China	L1
MF326989	IA/2014/ISU-2	2014	USA	L1
KT257997	ISU86	2014	USA	L1
KT257992	ISU73	2014	USA	L1
KT257975	ISU28	2014	USA	L1
KT258006	SDSU58	2014	USA	L1

KT257956	103837	2014	USA	L1
MN046228	HeNXX-2014-12	2014	China	L1
KR534894	OH155-2015	2015	USA	L1
KU950371	HENXC-4	2015	China	L1
KU950374	HENZMD-9	2015	China	L1
KX900392	HENJY-2	2015	China	L1
MF326987	IA/2015/NADC36	2015	USA	L1
MF326996	IA/2015/ISU-9	2015	USA	L1
MF326997	IA/2015/ISU-10	2015	USA	L1
MF326999	NC/2015/ISU-12	2015	USA	L1
MF327000	IA/2015/ISU-13	2015	USA	L1
MF327001	IA/2015/ISU-14	2015	USA	L1
MF326986	IA/2015/NADC35	2015	USA	L1
KX980392	SDhz1512	2015	China	L1
KX815411	15HEB1	2015	China	L1
KX815415	15HEN4	2015	China	L1
MN073090	PRR223341-S23-L001	2015	USA	L1
MN073091	PRR223343-S24-L001	2015	USA	L1
MN073182	PRR22334-1	2015	USA	L1
MK860181	394-1	2015	USA	L1
KU512796	CA-2-P12	2015	South Korea	L1
KU512797	CA-2-P22	2015	South Korea	L1
KU512798	CA-2-P32	2015	South Korea	L1
KU512799	CA-2-P42	2015	South Korea	L1
KU512800	CA-2-P52	2015	South Korea	L1
KU512801	CA-2-P62	2015	South Korea	L1
KU512802	CA-2-P72	2015	South Korea	L1
KU512803	CA-2-P82	2015	South Korea	L1
KU512804	CA-2-P92	2015	South Korea	L1
KU512805	CA-2-P100	2015	South Korea	L1
KU523366	WUH5	2015	China	L1
MF326998	NC/2015/ISU-11	2015	USA	L1
KX815413	15HEN1	2015	China	L1
KX815419	15JX1	2015	China	L1
KX815423	15LN1	2015	China	L1
MN073085	018560PRRS-S9-L001	2015	USA	L1
MF375260	SD-A19	2015	China	L1
KX758250	FJXS15	2015	China	L1
KX815432	15ZJ1	2015	China	L1
KT945017	HNjz15	2015	China	L1
KX815428	15SC3	2015	China	L1
KX815425	15LN3	2015	China	L1
MF375261	SC-d	2015	China	L1
KY053458	SDYG1606	2016	China	L1

MH651742	SDQD-1604	2016	China	L1
KX192112	NCV-13	2016	USA	L1
KX192113	NCV-16	2016	USA	L1
KX192114	NCV-17	2016	USA	L1
KX192115	NCV-21	2016	USA	L1
KX192116	NCV-23	2016	USA	L1
KX192117	NCV-24	2016	USA	L1
KX192118	NCV-25	2016	USA	L1
KX192119	NCV-26	2016	USA	L1
MN073093	PRR21032-S1-L001	2016	USA	L1
MN073094	brian27950-S16-L001	2016	USA	L1
MN175677	PRRSV2/USA/Lab3	2016	USA	L1
MH588710	SDbz16-2	2016	China	L1
MH651740	HNJYH-1606	2016	China	L1
MH651736	CY1-1604	2016	China	L1
MH651743	SD-1602	2016	China	L1
MF196906	SCnj16	2016	China	L1
MF766474	HeN1601	2016	China	L1
KY363991	CA-2-MP110	2016	South Korea	L1
KY363992	CA-2-MP120	2016	South Korea	L1
KX766379	HNhx	2016	China	L1
MN046240	SX1-1607	2016	China	L1
MN046241	SX2-1607	2016	China	L1
MN073087	018561PRRS-S10-L001	2016	USA	L1
KY495780	JX/CH/2016	2016	China	L1
KY495781	SH/CH/2016	2016	China	L1
MN073103	014737Fib-S5-L001	2016	USA	L1
MN073104	014737lu1A-S1-L001	2016	USA	L1
MN073105	014737lu2A-S2-L001	2016	USA	L1
MN073106	014737luC-S4-L001	2016	USA	L1
MN073107	014737Ton-S6-L001	2016	USA	L1
MN073108	014737luB-S3-L001	2016	USA	L1
MN073109	br42321BC-S13-L001	2016	USA	L1
MN073110	PRR41505-S4-L001	2016	USA	L1
MN073114	52335PRRS-S10-L001	2016	USA	L1
MN073115	5988810PRRS-S5-L001	2016	USA	L1
MH651737	CY2-1604	2016	China	L1
KX758249	FJWQ16	2016	China	L1
MH651744	SD53-1603	2016	China	L1
MH651745	SD99-1606	2016	China	L1
MN046222	HLJ-80	2016	China	L1
MH651746	SDQZ-1609	2016	China	L1
MH651739	HBFL-1604	2016	China	L1
KY041782	HENXX-8	2016	China	L1

MH651741	LNCH-1604	2016	China	L1
MH651748	TJZH-1607	2016	China	L1
MH651738	HNJYF-1606	2016	China	L1
MF526964	USA/IN_Purdue/1490-LN/2017	2017	USA	L1
MG913987	LNWK130	2017	China	L1
MH068878	SD17-38	2017	China	L1
MH121061	SD17-36	2017	China	L1
MH167388	QHD3	2017	China	L1
MG914067	SCcd17	2017	China	L1
MN073092	9982R-S5-L001	2017	USA	L1
MN073097	5606R-S6-L001	2017	USA	L1
MN073098	PRR312824-S16-L001	2017	USA	L1
MN073100	PRR312826-S18-L001	2017	USA	L1
MF526965	USA/IN_Purdue/14067/2017	2017	USA	L1
MN046224	HEB-108	2017	China	L1
MN073086	1924R-S2-L001	2017	USA	L1
MN073088	1923R-S1-L001	2017	USA	L1
MN073089	9337R-S4-L001	2017	USA	L1
MN073095	20170210381-S2-L001	2017	USA	L1
MN073113	PRR312823-S15-L001	2017	USA	L1
MN073118	PRR027981-S25-L001	2017	USA	L1
MN073119	5381R-S5-L001	2017	USA	L1
MN073120	5383R-S7-L001	2017	USA	L1
MN073125	PRR312827-S19-L001	2017	USA	L1
MN073111	PRR027983-S27-L001	2017	USA	L1
MN073112	PRR027984-S28-L001	2017	USA	L1
MG844181	HB17A	2017	China	L1
MH500776	NADC30-2017	2017	China	L1
MG687491	QHD1	2017	China	L1
MK396376	GDsf1707	2017	China	L1
MK396377	GDsf1710	2017	China	L1
MK396378	GDsf1711	2017	China	L1
MN073123	PRR312821-S13-L001	2017	USA	L1
MN073124	PRR312822-S14-L001	2017	USA	L1
MH167387	QHD2	2017	China	L1
MG860516	LNWK96	2017	China	L1
MH078490	SCN17	2017	China	L1
MG011719	FJDJQ-2017	2017	China	L1
MN073096	PRRSV-2018014208-S12	2018	USA	L1
MN073102	7705R-S1	2018	USA	L1
MN073174	PRRSV-2018017613-S8	2018	USA	L1
MK450333	CH-WH-2019-1	2018	China	L1
MK202794	FJ0908	2018	China	L1
MH370474	CH/2018/NCV-Anheal-1	2018	China	L1

MN046243	HLJ-DZD4-1805	2018	China	L1
MN073084	2385R-S13	2018	USA	L1
MK396379	GDsf1802	2018	China	L1
MK396380	GDsf1804	2018	China	L1
MN046230	HLJWK108-1711	2018	China	L1
MN073081	0752R-S4	2018	USA	L1
MN073083	7498R-S10	2018	USA	L1
MK396381	GDsf1806	2018	China	L1
MK396382	GDsf1807	2018	China	L1
MK396383	GDsf1808	2018	China	L1
MK396384	GDsf1809	2018	China	L1
MN073172	1504R-S1	2018	USA	L1
MN073122	6950-1R-S2	2018	USA	L1
MK796164	IA76950-WT	2018	USA	L1
MN046229	HeB-239	2018	China	L1
MN073127	7684R-S11	2018	USA	L1
MK144543	SCya18	2018	China	L1
DQ176019	MN184A	na	USA	L1
EF484031	MN184	na	USA	L1
EF488739	MN184C	na	USA	L1
DQ176020	MN184B	na	USA	L1
GQ914997	SD1-100	na	China	L1
JX258843	SD23983	na	USA	L1
GU232736	TP P60	na	China	L1
GU232737	TP P90	na	China	L1
KF287133	HK2	2003	China	L3
KF287138	HK11	2004	China	L3
KF287142	HK15	2004	China	L3
KF287137	HK9	2004	China	L3
KF287135	HK6	2004	China	L3
JQ743666	QY2010	2010	China	L3
JN662424	GM2	2011	China	L3
JQ308798	QYYZ	2011	China	L3
KY373215	HiNZWQ	2014	China	L3
KU978619	GD-KP	2015	China	L3
KX621003	GDsg	2015	China	L3
KX689233	XJzx1-2015	2015	China	L3
KT945018	HNyc15	2015	China	L3
MH236426	ZJnb16-2	2016	China	L3
KY745901	GDYDZZZ	2016	China	L3
MF196905	SCcd16	2016	China	L3
MK144542	GZgy17	2017	China	L3
MH324400	SCya17	2017	China	L3
MG011718	FJLIUY-2017	2017	China	L3

MN046242	LN-DB87	2018	China	L3
KP998476	FJFS	na	China	L3
AB288356	EDRD-1	1992	Japan	L4
KU131564	SD95-47_P83	1995	USA	L5
KC469618	SD95-21	1995	USA	L5
KC862576	DK-1997-19407B	1997	Denmark	L5
AF325691	NVSL_97-7985_IA_1-4-2	1997	USA	L5
NC_001961	16244B	1997	USA	L5
AY585241	PL97-1	1997	South Korea	L5
AY612613	PL97-1/LP1	1997	South Korea	L5
KU131562	ND99-14_P83	1999	USA	L5
KU131567	ND99-14_P84	1999	USA	L5
KU131569	ND99-14_P100	1999	USA	L5
KY348851	43807-00	2000	USA	L5
KY348853	3805-00	2000	USA	L5
KU131561	SD02-10_P83	2002	USA	L5
FJ899592	Clone20	2003	China	L5
KC862584	DK-2003-2-3	2003	Denmark	L5
KF287141	HK14	2004	China	L5
KC862578	DK-2004-1-7-Pl	2004	Denmark	L5
KU131559	SD04-89_P83	2004	USA	L5
KC862585	DK-2004-2-1	2004	Denmark	L5
JN864948	DY	2007	China	L5
KC862582	DK-2008-10-1-3	2008	Denmark	L5
KC862581	DK-2010-10-2-1	2010	Denmark	L5
KC862580	DK-2010-10-7-1	2010	Denmark	L5
KC862583	DK-2010-10-4-1	2010	Denmark	L5
KF183946	DK-2010-10-13-1	2010	Denmark	L5
JQ087873	A2MC2-2010	2010	USA	L5
KC862577	DK-2011-030311-1	2011	Denmark	L5
KF183947	DK-2011-88005-A8-Pl	2011	Denmark	L5
JX857698	YN-2011	2011	China	L5
KF771273	GZ1101	2011	China	L5
KC862575	DK-2012-01-11-3	2012	Denmark	L5
KM514315	PRRSV-2_Hungary_102_2012	2012	Hungary	L5
KX462792	A2MC2-2012	2012	USA	L5
KC445138	HZ-31	2012	China	L5
KT257945	14-64	2014	USA	L5
KT257946	14-67	2014	USA	L5
KT257947	14-68	2014	USA	L5
KT257944	14-60	2014	USA	L5
KU318406	A2MC2-P90	2015	USA	L5
MN073134	PRR715665-S8-L001	2015	USA	L5
MN073135	PRR715666-S9-L001	2015	USA	L5

MN073133	7473PRRS-S3-L001	2016	USA	L5
MN073139	7471PRRS-S1-L001	2016	USA	L5
MN073136	397081R-S16-L001	2017	USA	L5
MN073137	397082R-S17-L001	2017	USA	L5
MN073140	lung23199-S4-L001	2017	USA	L5
MN073143	PRR146795SA-S1-L001	2017	USA	L5
MN073145	PRR1467916SA-S2-L001	2017	USA	L5
MN073146	PRR1467916SQ-S4-L001	2017	USA	L5
MN073147	PRRS35752R-S7-L001	2017	USA	L5
MN073148	PRR02760-S1-L001	2017	USA	L5
MN073149	PRR02760Q-S15-L001	2017	USA	L5
MN046233	Liaoning-2017-6	2017	China	L5
MN073130	4115R-S1	2018	USA	L5
MN073131	1041R-S1	2018	USA	L5
MN073132	4512R-S7-L001	2018	USA	L5
DQ217415	VR-2332-pVR-V7	na	USA	L5
KP998474	FJSD	na	China	L5
AF066183	RespPRRS MLV	na	USA	L5
DQ176021	VR2332 V7	na	USA	L5
DQ459471	S1	na	China	L5
EF484033	Ingelvac MLV	na	USA	L5
FJ524376	VR-2332-V7-HA-myc	na	USA	L5
FJ524377	VR-2332-pV7-myc	na	USA	L5
DQ056373	01NP1.2	na	Thailand	L5
AF159149	MLV ResPRRS/Repro	na	na	L5
AF176348	PA8	na	Canada	L5
AF331831	BJ-4	na	China	L5
AY150564	VR-2332	na	USA	L5
EU360128	rV68	na	China	L5
EU360129	rV63	na	China	L5
EU880441	GS2002	na	China	L5
EU880442	GS2003	na	China	L5
EU880443	GS2004	na	China	L5
FJ175687	PRRSV01	na	China	L5
FJ175688	PRRSV02	na	China	L5
FJ175689	PRRSV03	na	China	L5
AY457635	HN1	na	China	L5
EF153486	CC-1	na	China	L5
AF494042	P129	na	USA	L6
MN073129	41761R-S15-L001	2018	USA	L7
AF184212	SP	na	na	L7
DQ779791	Prime Pac	na	USA	L7
MK837936	NADC20	1996	USA	L8
JN654458	SDSU73	1996	USA	L8

AY262352	HB-2(sh)/2002	2002	China	L8
EU360130	HB-1/3.9	2002	China	L8
KF287132	HK1	2003	China	L8
KF287136	HK7	2004	China	L8
FJ536165	NB/04	2004	China	L8
KF287139	HK12	2004	China	L8
KF287140	HK13	2005	China	L8
KJ541663	GZ106	2005	China	L8
EU864232	SHB	2005	China	L8
KU131558	MN05-68_P83	2005	USA	L8
MF370557	FZ06A	2006	China	L8
EF641008	JXwn06	2006	China	L8
EU144079	SY0608	2006	China	L8
EU708726	JX143	2006	China	L8
EU860248	TJ	2006	China	L8
EU864233	TP	2006	China	L8
EU880432	JX2006	2006	China	L8
FJ797690	HN-HW	2006	China	L8
MF187956	rJXwn06	2006	China	L8
FJ950744	BJSY-1	2007	China	L8
JX317648	HV	2007	China	L8
JX512910	SRV07	2007	Viet Nam	L8
EF488048	pJX143	2007	China	L8
EU825723	BJ	2007	China	L8
EU825724	GD-825724	2007	China	L8
EU860249	NM1	2007	China	L8
EU864231	CG	2007	China	L8
EU880433	GD2007	2007	China	L8
EU880434	SX2007	2007	China	L8
EU880437	HN2007	2007	China	L8
FJ393456	07NM	2007	China	L8
FJ393457	07HEN	2007	China	L8
FJ393458	07HEBTJ	2007	China	L8
FJ393459	07BJ	2007	China	L8
FJ950747	BJSD	2007	China	L8
GQ374441	GDQJ	2007	China	L8
GU461292	AH0701	2007	China	L8
HM011104	BJSY07	2007	China	L8
JN387271	GDQY1	2007	China	L8
KX766378	HN07-1	2007	China	L8
FJ950745	BJBLZ	2007	China	L8
EU624117	XH-GD	2007	China	L8
GQ351601	BJ0706	2007	China	L8
HQ401282	Shaanxi-2	2007	China	L8

FJ950746	BJPG	2007	China	L8
GU454850	GDQY2	2007	China	L8
GU169411	08HuN	2008	China	L8
EU880431	GS2008	2008	China	L8
EU880435	YN2008	2008	China	L8
EU880436	XL2008	2008	China	L8
FJ889129	CBB-1-F3	2008	China	L8
FJ889130	CWZ-1-F3	2008	China	L8
GQ359108	SD-CXA/2008	2008	China	L8
GQ374442	GDBY1	2008	China	L8
GQ499193	JXM20	2008	China	L8
GQ499194	JXM40	2008	China	L8
GQ499195	JXM60	2008	China	L8
GQ499196	JXM80	2008	China	L8
HM853673	WUH3	2008	China	L8
KP771754	NVDC-HeB-2008	2008	China	L8
KP771755	NVDC-CQ-2008	2008	China	L8
KP771779	NVDC-NM-2008	2008	China	L8
KT033733	QH-1(sh)	2008	China	L8
KU201579	QH-08	2008	China	L8
HM016158	JN-HS	2008	China	L8
KX650082	RVB-581	2008	China	L8
HQ315836	NT0801	2008	China	L8
KJ523894	NT0801_P10	2008	China	L8
GU168569	08SDWF	2008	China	L8
GU232735	KP	2008	China	L8
GU232738	YN9	2008	China	L8
HM016159	ZP-1	2009	China	L8
JF800911	ZCYZ	2009	China	L8
FJ895329	SX2009	2009	China	L8
GU143913	GD-143913	2009	China	L8
HM189676	HLJHL	2009	China	L8
HQ315835	BB0907	2009	China	L8
HQ315837	SY0909	2009	China	L8
HQ843178	HLJ-09	2009	China	L8
HQ843179	HLM-09	2009	China	L8
HQ843180	SD-09	2009	China	L8
HQ843181	SX-09	2009	China	L8
JF268672	09SC	2009	China	L8
JF268673	09HUN1	2009	China	L8
JF268676	09BJ	2009	China	L8
JF268678	09SD	2009	China	L8
JF268679	09HEB	2009	China	L8
JF268680	09HEN2	2009	China	L8

JF268682	09HUB1	2009	China	L8
JF268683	09HUB2	2009	China	L8
JF268684	09HEN1	2009	China	L8
GU168568	09HUB5	2009	China	L8
GU168567	09HUB7	2009	China	L8
JF268674	09HUN2	2009	China	L8
JF268677	09DB1	2009	China	L8
JF268681	09DB2	2009	China	L8
KJ523895	NT0801_P30	2009	China	L8
JF268675	09JS	2009	China	L8
JQ804986	JXA1-P170	2009	China	L8
KC422725	JXA1-P100	2009	China	L8
KC422726	JXA1-P110	2009	China	L8
KC422727	JXA1-P120	2009	China	L8
KC422728	JXA1-P130	2009	China	L8
KC422729	JXA1-P140	2009	China	L8
KC422730	JXA1-P150	2009	China	L8
KC422731	JXA1-P160	2009	China	L8
JF748717	YD	2009	China	L8
JN836553	SCwhn09CD	2009	China	L8
JX192633	10GZ-GD	2010	China	L8
JX317649	JX	2010	China	L8
KF735060	HP/Thailand/19500LL/2010	2010	Thailand	L8
JX215553	10HD-GD	2010	China	L8
JN387272	GDQY1VP65	2010	China	L8
JN387273	GDQY1VP80	2010	China	L8
JQ663540	10-10JX	2010	China	L8
JQ663541	10-10BJ-1	2010	China	L8
JQ663542	10-10BJ-3	2010	China	L8
JQ663543	10-10BJ-2	2010	China	L8
JQ663544	10-10BJ-4	2010	China	L8
JQ663545	10-10BJ-5	2010	China	L8
JQ663546	10-10FUJ-1	2010	China	L8
JQ663547	10-10FUJ-2	2010	China	L8
JQ663548	10-10FUJ-3	2010	China	L8
JQ663549	10-10FUJ-4	2010	China	L8
JQ663550	10-10FUJ-5	2010	China	L8
JQ663551	10-10HEB-1	2010	China	L8
JQ663552	10-10HEB-2	2010	China	L8
JQ663553	10-10HEB-3	2010	China	L8
JQ663555	10-10SD	2010	China	L8
JQ663556	10-10QN	2010	China	L8
JQ663557	10-LW1-13	2010	China	L8
JQ663558	10-10GX-1	2010	China	L8

JQ663559	10-10GX-2	2010	China	L8
JQ663560	10-10GX-3	2010	China	L8
JQ663561	10-10GX-4	2010	China	L8
JQ663562	10-10GX-5	2010	China	L8
JQ663563	10-LW2-6	2010	China	L8
JQ663564	10-LW3-7	2010	China	L8
JQ663565	10-10LW5-1	2010	China	L8
JQ663566	10-LW6-6	2010	China	L8
JQ663567	10-LW7-1	2010	China	L8
JQ663568	10-LW8-1	2010	China	L8
JN626287	BH58/10	2010	Laos	L8
JF796180	FS	2010	China	L8
JX192637	10SJ-GD	2010	China	L8
JX192638	10SS-GD	2010	China	L8
JX192634	10FS-GD	2010	China	L8
JX192636	10BY-GD	2010	China	L8
JX215552	10QY-GD	2010	China	L8
JX192639	10ZQ-GD	2010	China	L8
KJ523896	NT0801_P50	2010	China	L8
KJ855518	Shanxi-6	2010	China	L8
JF748718	DC	2010	China	L8
JX912249	GX1003	2010	China	L8
JX192635	10FS1-GD	2010	China	L8
JX192632	10HN-GD	2010	China	L8
JN387274	GDQY1VP100	2011	China	L8
JQ326271	WUH4	2011	China	L8
JQ715698	NVDC-JS2-2011	2011	China	L8
KC527830	GD-2011	2011	China	L8
KF751237	BJ1102	2011	China	L8
KP771746	NVDC-CQ1-2011	2011	China	L8
KP771748	NVDC-BJ2-2011	2011	China	L8
KP771749	NVDC-HeB1-2011	2011	China	L8
KP771765	NVDC-HeB2-2011	2011	China	L8
KP771766	NVDC-GD-2011	2011	China	L8
KP771767	NVDC-YN-2011	2011	China	L8
KP771774	NVDC-CQ3-2011	2011	China	L8
KP771778	NVDC-BJ1-2011	2011	China	L8
KP793736	GD-HD	2011	China	L8
JX878380	SDA3	2011	China	L8
JX215551	11FS11-GD	2011	China	L8
JX215554	11FS12-GD	2011	China	L8
JX235366	11SH1-GD	2011	China	L8
JX235370	11GZ-GD	2011	China	L8
JX679179	HH08	2011	China	L8

KF751238	LN1101	2011	China	L8
JX880029	NJ-1106	2011	China	L8
KJ523897	NT0801_P80	2011	China	L8
JX878379	SDA2	2011	China	L8
JX217036	11NZ-GD	2011	China	L8
JX235367	11XX-GD	2011	China	L8
JX235365	11SH-GD	2011	China	L8
JQ715697	NVDC-GD2-2011	2011	China	L8
JX087437	SD16	2012	China	L8
KF678434	SH1211	2012	China	L8
MF689000	HeN1201	2012	China	L8
JQ663554	10-10JL	2012	China	L8
JX177644	JL-04/12	2012	China	L8
KF815525	XJu-1	2012	China	L8
KP771747	NVDC-CQ1-2012	2012	China	L8
KP771757	NVDC-BJ8-2012	2012	China	L8
KP771758	NVDC-BJ7-2012	2012	China	L8
KP771759	NVDC-BJ6-2012	2012	China	L8
KP771760	NVDC-BJ5-2012	2012	China	L8
KP771761	NVDC-BJ4-2012	2012	China	L8
KP771763	NVDC-BJ2-2012	2012	China	L8
KP771764	NVDC-BJ1-2012	2012	China	L8
KP771769	NVDC-SD1-2012	2012	China	L8
KP771770	NVDC-HuN-2011	2012	China	L8
KP771771	NVDC-HeN-2012	2012	China	L8
KP771772	NVDC-HeB2-2012	2012	China	L8
KP771775	NVDC-CQ3-2012	2012	China	L8
KP771776	NVDC-CQ2-2012	2012	China	L8
KP771777	NVDC-CQ4-2012	2012	China	L8
KP771756	NVDC-BJ9-2012	2012	China	L8
KP771762	NVDC-BJ3-2012	2012	China	L8
KP771768	NVDC-SD2-2012	2012	China	L8
KM189443	SC2012	2012	China	L8
KP771773	NVDC-HeB1-2012	2012	China	L8
MF766470	HeN1301	2013	China	L8
KJ002451	HeNan-A1	2013	China	L8
KJ002452	HeNan-A2	2013	China	L8
KJ019330	Henan-A3	2013	China	L8
KJ534539	Henan-A4	2013	China	L8
KJ534540	Henan-A5	2013	China	L8
KJ534541	Henan-A6	2013	China	L8
KJ534542	Henan-A7	2013	China	L8
KJ534543	Henan-A8	2013	China	L8
KJ546412	HeNan-A9	2013	China	L8

KJ609516	MY-486	2013	China	L8
KJ609517	MY-376	2013	China	L8
KP771740	NVDC-SXJC-2013	2013	China	L8
KP771742	NVDC-HBCZ-2013	2013	China	L8
KP771744	NVDC-HeB2-2013	2013	China	L8
KP771745	NVDC-HeB1-2013	2013	China	L8
KP771750	NVDC-MD2-2013	2013	China	L8
KP771751	NVDC-MD1-2013	2013	China	L8
KP771752	HEB_20130008-14	2013	China	L8
KP771753	HEB_20130008-13	2013	China	L8
KP890337	BJ-F20	2013	China	L8
KP890338	BJ-F40	2013	China	L8
KP890339	BJ-F60	2013	China	L8
KP890340	BJ-F80	2013	China	L8
KP890341	BJ-F110	2013	China	L8
KP890342	BJ-F150	2013	China	L8
KT180169	XF1129	2013	China	L8
KT351739	HLJA1	2013	China	L8
KT351740	HLJB1	2013	China	L8
KJ591659	HEB-2013	2013	China	L8
KP771741	NVDC-SDXX-2013	2013	China	L8
KP771743	NVDC-BJPG-2013	2013	China	L8
KT022072	HNyc13	2013	China	L8
KU950373	HENZK-1	2014	China	L8
KJ819934	Henan-A12	2014	China	L8
KJ819936	Henan-A14	2014	China	L8
KM453698	BB0907-s34	2014	China	L8
KM453699	BB0907-F44	2014	China	L8
KP742986	TJbd14-1	2014	China	L8
KP742987	TJbd14-2	2014	China	L8
KP771735	NVDC-SHH02-2014	2014	China	L8
KP771736	NVDC-shh01-2014	2014	China	L8
KP771737	NVDC-SD6-2014	2014	China	L8
KP771780	NVDC-13SXJC-2014	2014	China	L8
KP771783	NVDC-R224-2014	2014	China	L8
KP771784	NVDC-SD4-2014	2014	China	L8
KP780881	14LY01-FJ	2014	China	L8
KP780882	14LY02-FJ	2014	China	L8
KT819203	SCwhn14DY	2014	China	L8
KY373217	SDZZ	2014	China	L8
KY373218	SXF105	2014	China	L8
KY373216	AHBZ	2014	China	L8
KP771739	NVDC-SC1-2014	2014	China	L8
KP771781	NVDC-HuNCS-2014	2014	China	L8

KT445876	HNP5	2014	China	L8
KM261784	HB2014001	2014	China	L8
KT022071	HNxa14	2014	China	L8
MF669720	GD1404	2014	China	L8
KP771738	NVDC-SD1-2014	2014	China	L8
MN046221	2014-81	2014	China	L8
MN046227	HeNXX-2014-9	2014	China	L8
MF124329	GD1404	2014	China	L8
KP330232	HUN-2014	2014	China	L8
MF669722	ZJXS1412	2014	China	L8
KM000066	NMG2014	2014	China	L8
KJ819935	Henan-A13	2014	China	L8
KT257953	14-96	2014	USA	L8
KT257950	14-79	2014	USA	L8
KT257952	14-95	2014	USA	L8
KT257948	14-76	2014	USA	L8
KP771782	NVDC-R38-2014	2014	China	L8
KU950375	HENZZ-8	2015	China	L8
MF766472	HeN1501	2015	China	L8
MF766473	HeN1502	2015	China	L8
KT358728	GZgy15-1	2015	China	L8
KX767091	GSWW/CHA 2015	2015	China	L8
KX815407	15GD1	2015	China	L8
KX815408	15GD2	2015	China	L8
KX815409	15GD3	2015	China	L8
KX815410	15GD4	2015	China	L8
KX815414	15HEN3	2015	China	L8
KX815416	15HUN1	2015	China	L8
KX815417	15HUN2	2015	China	L8
KX815418	15HUN3	2015	China	L8
KX815424	15LN2	2015	China	L8
KX815429	15SN1	2015	China	L8
KX815430	15SN2	2015	China	L8
KX815431	15SN3	2015	China	L8
KX815412	15HEB3	2015	China	L8
KU950370	HENPDS-2	2015	China	L8
KX815420	15JX2	2015	China	L8
KX815421	15JX3	2015	China	L8
KX815427	15SC2	2015	China	L8
KX815433	15ZJ2	2015	China	L8
KX815434	15ZJ3	2015	China	L8
KX815422	15JX4	2015	China	L8
KX815426	15SC1	2015	China	L8
MN046237	InterMo-2015-2	2015	China	L8

MF669721	HZL1501	2015	China	L8
MN073166	PRR80785GJ-S21-L001	2015	USA	L8
MN073167	PRR80785GD-S22-L001	2015	USA	L8
MN073181	PRR80785	2015	USA	L8
MN073153	PRR715664-S10-L001	2015	USA	L8
KY761966	FZ16A	2016	China	L8
MF818049	SC/NJ 2016	2016	China	L8
MH651747	SDZC-1609	2016	China	L8
KX357708	QTX	2016	China	L8
KY290748	HENXX-9	2016	China	L8
MN046234	SDJM-1602	2016	China	L8
MN046238	HN-1603	2016	China	L8
KX980393	SDlz1601	2016	China	L8
MF526896	GDQYQC2	2016	China	L8
MN073168	562482PRRS-S4-L001	2016	USA	L8
MN073155	PRR3-S3-L001	2016	USA	L8
MH404256	SD17	2017	China	L8
MF770574	17-ZJ-HZ	2017	China	L8
MN046231	Anhui-2017-109	2017	China	L8
MN046235	Gansu-2017-51	2017	China	L8
MN046236	Sichuan-2017-117	2017	China	L8
MK759853	XJ17-5	2017	China	L8
MK906026	JSTZ1712-12	2017	China	L8
MH663433	HNRZ	2017	China	L8
MN073156	PRRSV-2017083451-S10	2017	USA	L8
MN073158	2017021039-S12-L001	2017	USA	L8
MN073159	lung249621-S5-L001	2017	USA	L8
MN073161	9985R-S8-L001	2017	USA	L8
MN073162	9981R-S4-L001	2017	USA	L8
MN073164	serum24962-S7-L001	2017	USA	L8
MN073165	9983R-S6-L001	2017	USA	L8
MN073169	6660R-S5-L001	2017	USA	L8
MN073170	9986R-S9-L001	2017	USA	L8
MN073171	9987R-S10-L001	2017	USA	L8
MN046223	HLJ-DZD1-1804	2018	China	L8
MN046232	JS3-1805	2018	China	L8
MN046239	HLJ-YC8	2018	China	L8
MK450365	CH-YY	2018	China	L8
MN073157	3451R-S10-L001	2018	USA	L8
MK796165	IA70388-R	2018	USA	L8
EF635006	HUN4	na	China	L8
FJ394029	07QN	na	China	L8
KT804696	FJYR	na	China	L8
EF112445	JXA1	na	China	L8

EF112446	HUB2	na	China	L8
EF112447	HEB1	na	China	L8
EF517962	HuN	na	China	L8
EU097706	NX06	na	China	L8
EU097707	BJsy06	na	China	L8
EU106888	SHH	na	China	L8
EU109502	LN	na	China	L8
EU109503	GD-109503	na	China	L8
EU200961	Jiangxi-3	na	China	L8
EU200962	Henan-1	na	China	L8
EU236259	HPBEDV	na	na	L8
EU678352	WUH2	na	China	L8
FJ548854	JXA1-P10	na	China	L8
FJ548855	JXA1-P15	na	China	L8
GQ475526	JXM100	na	China	L8
GQ857656	SX-1	na	China	L8
HM214913	GX09-16	na	China	L8
HM214914	GX09-29	na	China	L8
HM214915	GX09-32	na	China	L8
JQ309822	GX10-42	na	China	L8
KP998475	FJE1	na	China	L8
KP998479	FJOU	na	China	L8
KR149645	JXja15	na	China	L8
KU215417	15LY02-FJ	na	China	L8
KY488470	GDJM	na	China	L8
KY488471	GDGZ	na	China	L8
KY488473	GDZQ	na	China	L8
KY488474	HNHK2	na	China	L8
KY488475	HNHK1	na	China	L8
KY488476	GDHY	na	China	L8
KY488477	GDHZ	na	China	L8
KY488478	GDQY	na	China	L8
KY488479	GDSG	na	China	L8
KY498542	GDST	na	China	L8
EU187484	WUH1	na	China	L8
KU215416	15LY01-FJ	na	China	L8
EF075945	HUB1	na	China	L8
JQ309823	GX10-48	na	China	L8
FJ548851	JXA1-P45	na	China	L8
FJ548852	JXA1-P70	na	China	L8
FJ548853	JXA1-P80	na	China	L8
JQ955657	GX1001	na	China	L8
KP998477	FJCH	na	China	L8
KP998478	FJZH	na	China	L8

JQ955658	GX1002	na	China	L8
KY488472	GDMM	na	China	L8
EU262603	Em2007	na	China	L8
GQ330474	APRRS	na	China	L8
AY032626	CH-1a	na	China	L8
EU880438	CH2002	na	China	L8
EU880439	CH2004	na	China	L8
EU880440	CH2003	na	China	L8
KX510269	TJnh1501	na	China	L8
MF772778	GDzj	na	China	L8
AY424271	JA142	na	na	L8
DQ988080	Ingelvac ATP	na	USA	L8
AY545985	NVSL_97-7895	na	USA	L8
KU131565	SD95-10_P83	1995	USA	L9
KY348847	1692-98	1998	USA	L9
KU131563	SD98-163_P83	1998	USA	L9
KY348852	46517-00	2000	USA	L9
KY348850	21599-00	2000	USA	L9
HQ699067	NC16845	2006	USA	L9
