# Supplementary figures and images for: A Novel Motif in the 3′-UTR of PRRSV-2 Is Critical for Viral Multiplication and Contributes to Enhanced Replication Ability of Highly Pathogenic or L1 PRRSV
Source: Viruses. 2022 Jan 18;14(2):166. doi: 10.3390/v14020166 (PMC8875199; doi:10.3390/v14020166)

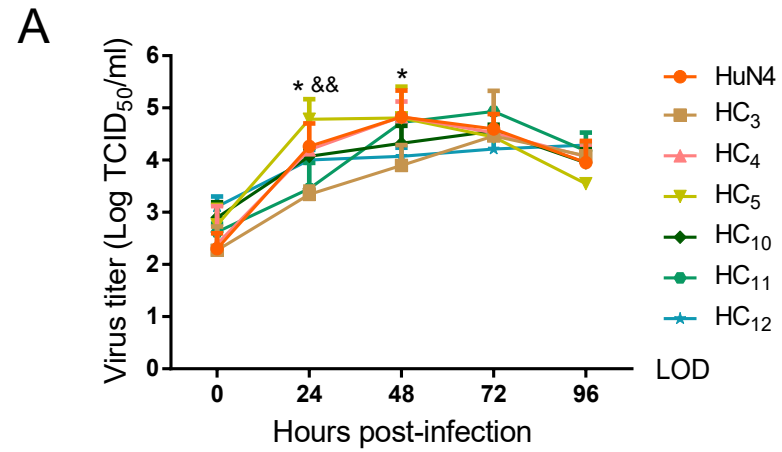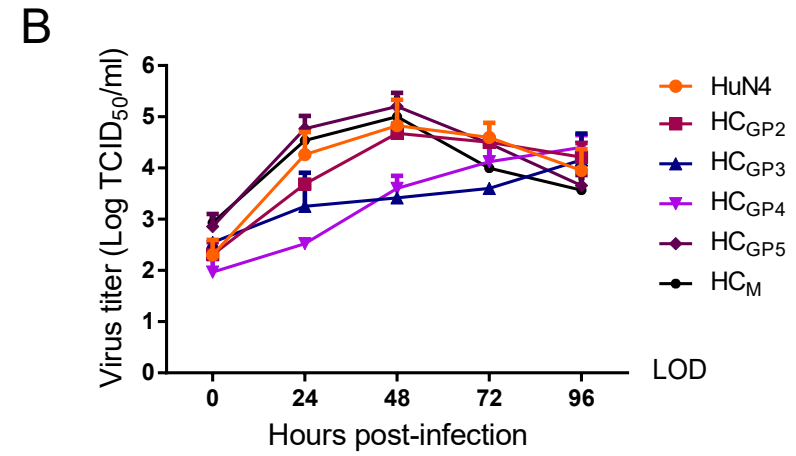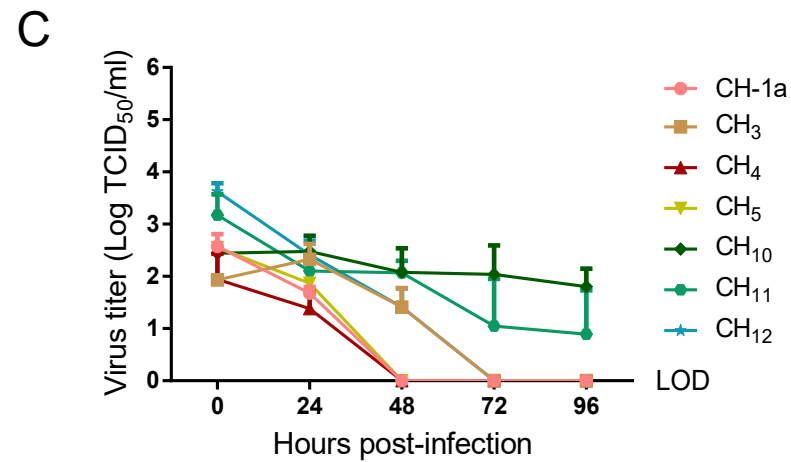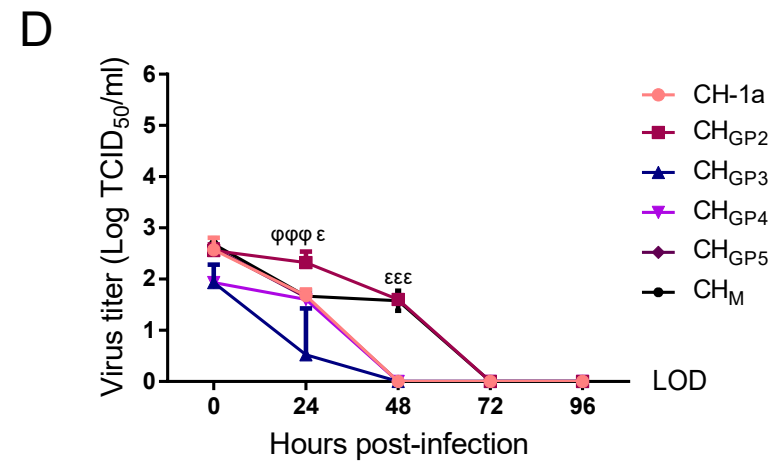

Supplement: Supplementary file 1 [file viruses-14-00166-s001.zip › PDF/Fig S1. The comparison of replication efficiency between parental and chimeric viruses in PAMs..pdf]

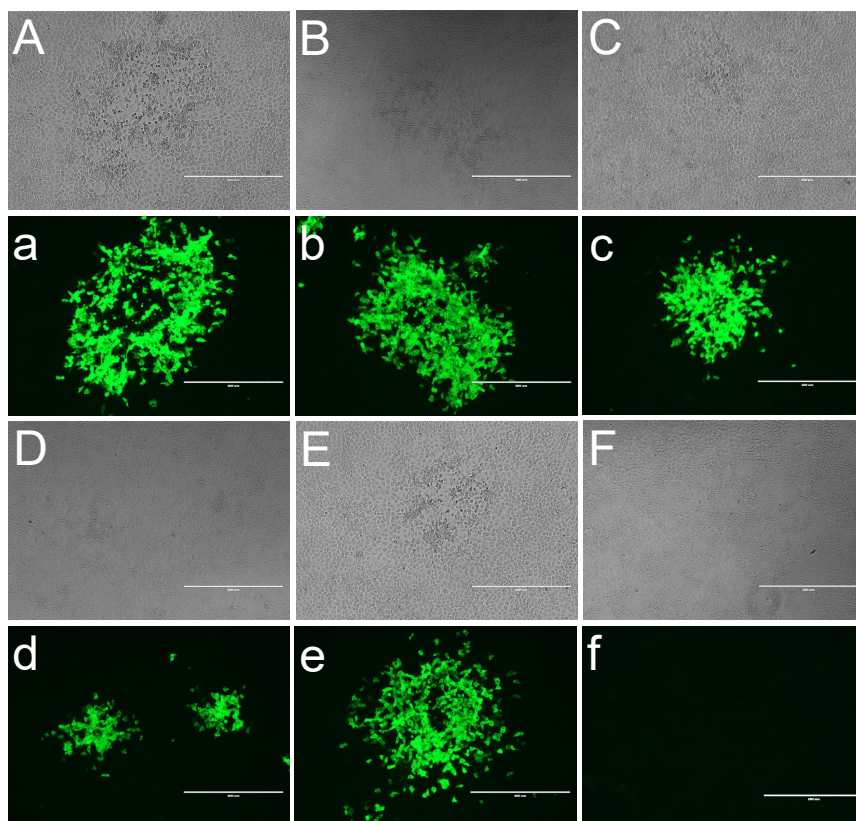

G

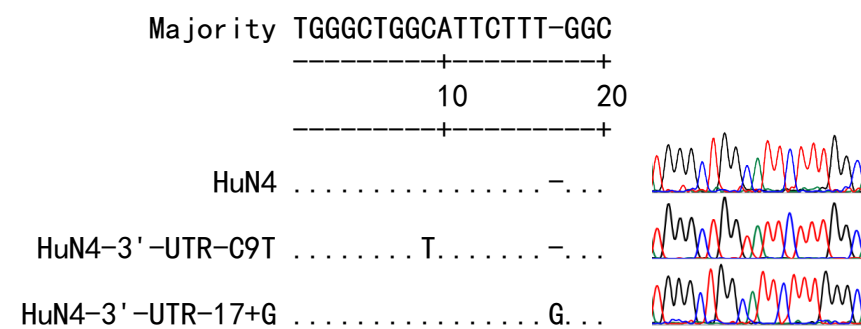

H

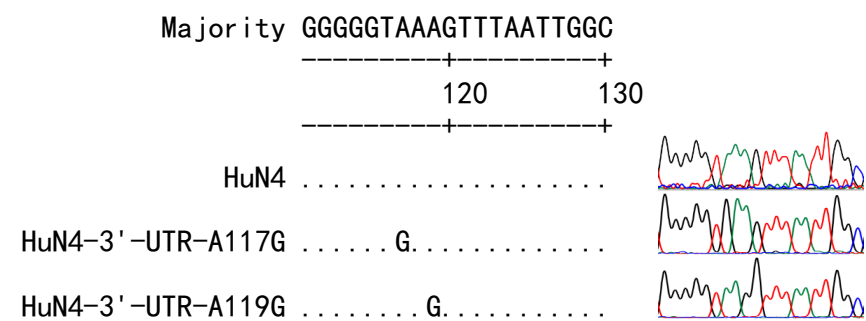

Supplement: Supplementary file 1 [file viruses-14-00166-s001.zip › PDF/Fig S2. Identification of mutant viruses.pdf]

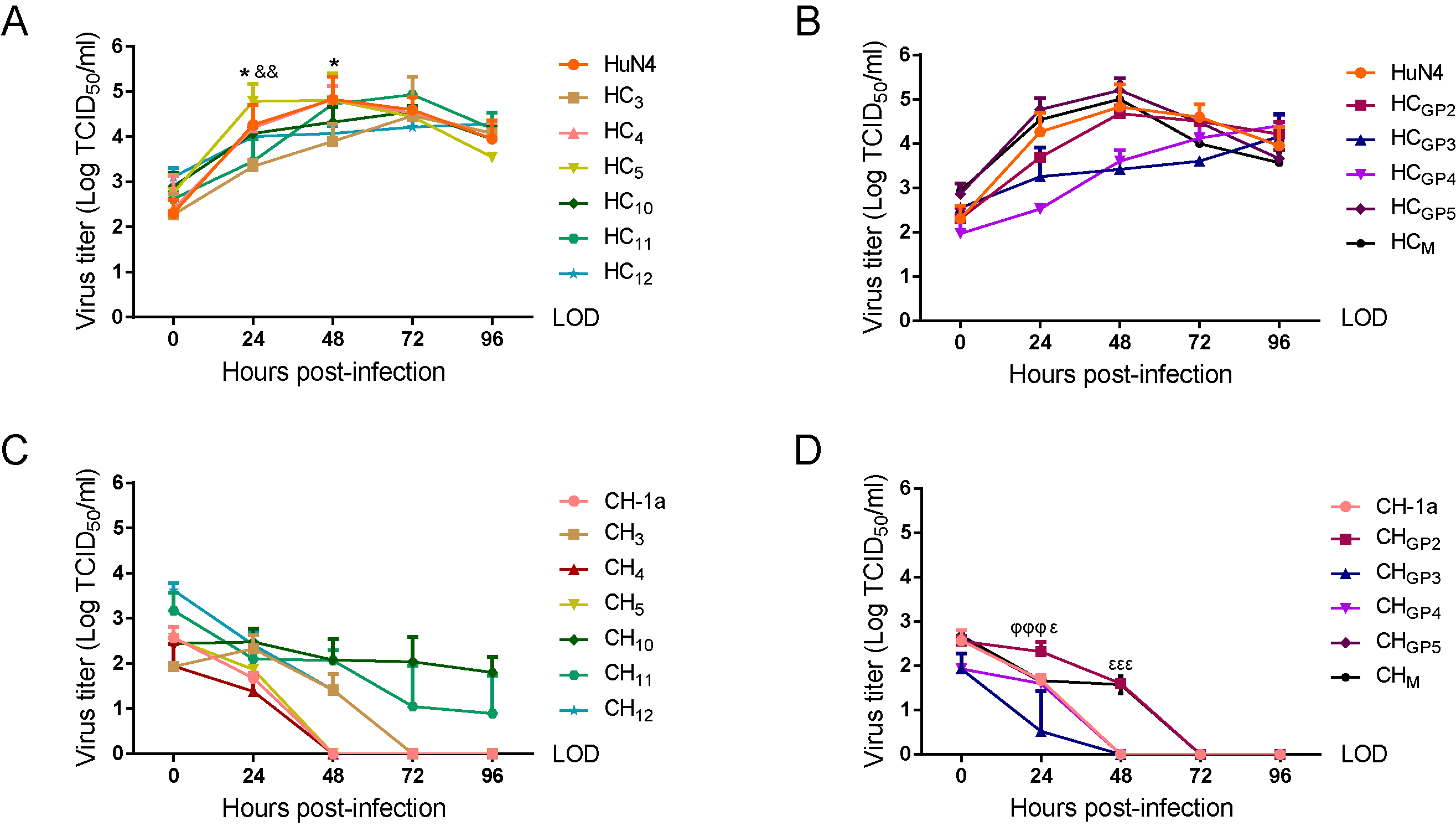

Supplement: Supplementary file 1 [file viruses-14-00166-s001.zip › Tiff/Fig S1. The comparison of replication efficiency between parental and chimeric viruses in PAMs..tif]

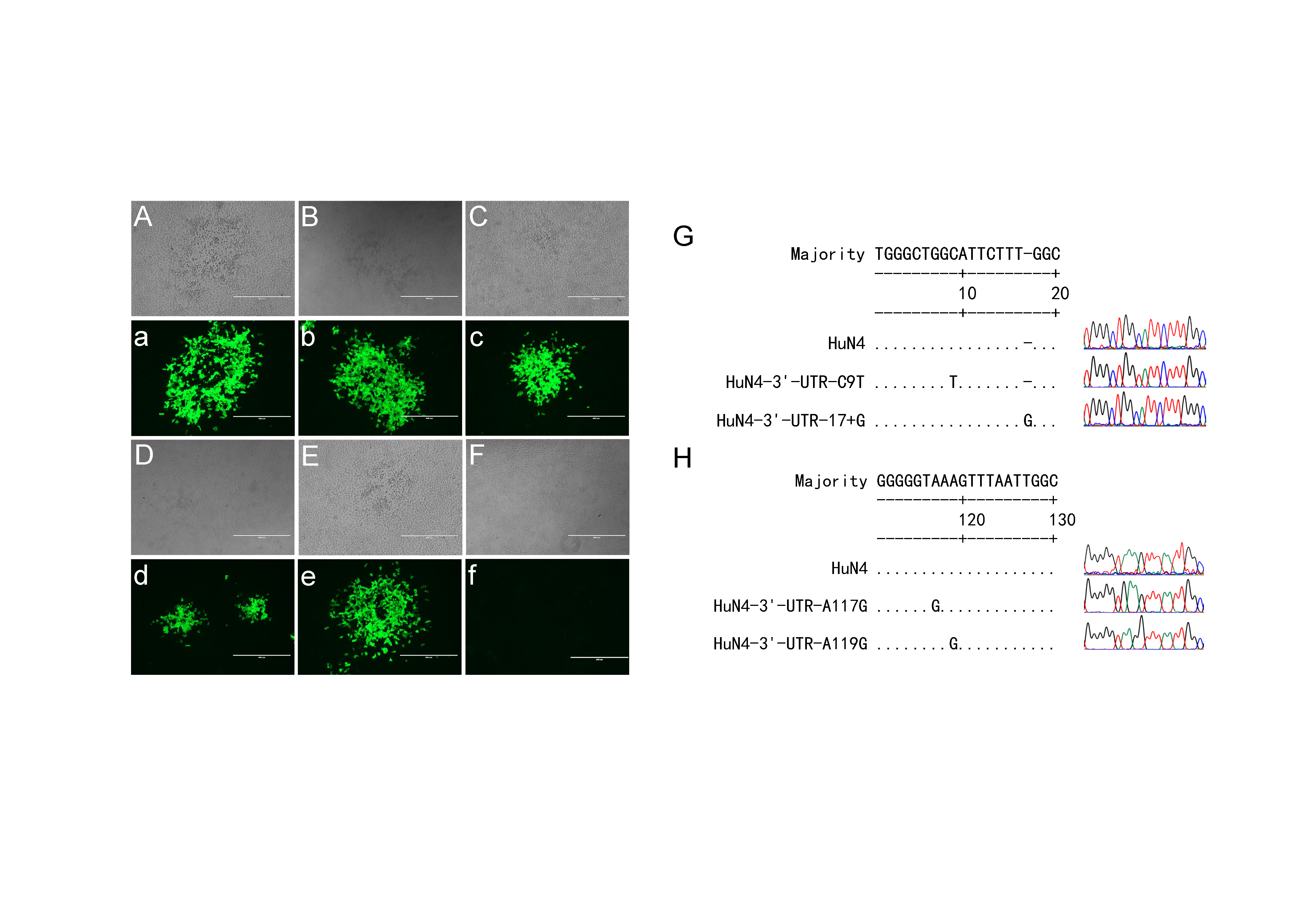

Supplement: Supplementary file 1 [file viruses-14-00166-s001.zip › Tiff/Fig S2. Identification of mutant viruses.tiff]
